# Speciation and Aqueous Dissolution of Macronutrients in Fire Ash: Variation across Ecosystems and the Effects on Nutrient Cycling

**DOI:** 10.1021/acs.est.4c07101

**Published:** 2024-12-24

**Authors:** Lingqun Zeng, Shyrill F. Mariano, Rixiang Huang, Carmen Sánchez-García, Cristina Santin, Jonay Neris, Kruthika Kumar, Chase K. Glenn, Omar El Hajj, Anita Anosike, Joseph O’Brien, Rawad A. Saleh

**Affiliations:** † Department of Environmental and Sustainable Engineering, University at Albany, 1400 Washington Avenue, Albany 12222, New York, United States; ‡ Centre for Wildfire Research, Department of Geography, Swansea University, Singleton Campus, Swansea SA2 8PP, U.K.; § Biodiversity Research Institute (IMIB; CSIC—Universidad de Oviedo—Principality of Asturias), Mieres 33600, Spain; ∥ Departamento de Biología Animal, Edafología y Geología, Universidad de La Laguna, La Laguna 38200, Spain; ⊥ School of Environmental, Civil, Agricultural and Mechanical Engineering, University of Georgia, Athens 30602, Georgia, United States; # USDA Forest Service Southern Research Station, Athens 30602, Georgia, United States

**Keywords:** terrestrial ecosystems, wildland fires, fire ash, smoke, macronutrients, speciation, X-ray absorption spectroscopy (XAS)

## Abstract

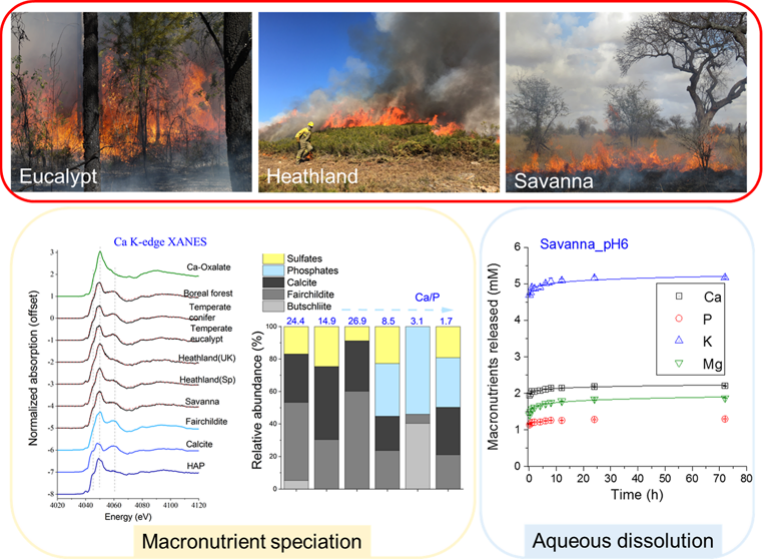

This study investigated the speciation and aqueous dissolution of macronutrients in fire ash from diverse ecosystems and speciation of ash and smoke from laboratory burning, exploring the variations and their causes. The speciation of phosphorus (P), calcium (Ca), and potassium (K) in fire ash from five globally distributed ecosystems was characterized by using X-ray absorption spectroscopy and sequential fractionation. Aqueous dissolution of the macronutrients was measured by batch experiments at acidic and alkaline pHs. The results showed that P existed mainly as Ca phosphates, Ca as double carbonates, calcite, and sulfates, and most K was associated with Ca carbonates. Mineralogy and the relative abundance of the species were primarily controlled by elemental stoichiometry and fire temperature. Differences in Ca and P speciation existed between ash and smoke from laboratory burning, possibly caused by the temperature difference and/or mass fractionation during burning. The rates, extents, and pH dependencies of macronutrient dissolution differed among macronutrients and depended on their speciation, with K being highly soluble and the P and Ca regulated by solution pH. The variability in ash macronutrient chemistry and ecosystem-specific fire ash loads resulted in varying loads and availability of individual macronutrient from fire among ecosystems. This study provides a mechanistic understanding of how fires transform the chemistry of macronutrients and affect macronutrient returns to soils across different ecosystems, which is essential for evaluating the disturbance to ecosystem nutrient cycling by fires.

## Abstract 2

This study revealed molecular-level chemistry of macronutrients in fire ash and their variations across terrestrial ecosystems, which were shown to affect the amounts and availability of individual macronutrient from fires.

## 1 Introduction

As terrestrial ecosystems develop and pedogenesis proceeds, the cycling of macronutrients is increasingly driven by biological processes—vegetation accumulates macronutrients in living and dead biomass,^1,2^ whose decomposition is primarily mediated by microbes. The microbe-mediated organic matter decomposition process influences soil chemical composition^3^ and is an important macronutrient source for plant uptake.^4^ Fires are a common and probably the most pervasive disturbance to most terrestrial ecosystems,^5^ and the cycling of macronutrients is among the many ecosystem processes changed by them. The burning of biomass alters the aboveground pools of macronutrients and their physicochemical forms,^6,7^ changing therefore their cycling. For example, the returning of aboveground macronutrients to soils will shift from a primarily biotic pathway to an abiotic one.^8^ Considering the importance of aboveground macronutrient pools in the overall ecosystem budget and soil nutrient composition, it is necessary to understand the chemical forms of macronutrients in fire burning products and their postfire fate and transport.

During wildland fires (including wildfire and prescribed fires), fuel biomass is burned, emitting particles and gases as smoke into the atmosphere and depositing solid ash on the ground. Herein, fire ash is defined as ground-deposited solids that contains both complete and incomplete burning products (ash and char, respectively), from all types of vegetation burning.^9^ Mineral macronutrients [i.e., calcium (Ca), potassium (K), magnesium (Mg), and phosphorus (P)] previously in the fuel biomass are partitioned into these burning products,^10,11^ the composition and elemental speciation of which largely determine their geochemical behaviors in postfire environments.

Previous studies focused primarily on characterizing the elemental composition and aqueous solubility of fire ash from different ecosystems and fire conditions.^12−14^ For example, varying water-soluble macronutrient contents among ash samples of different colors and fire severities have been observed, but the causes remain unclear.^12,13^ Other research investigated postfire macronutrient export via runoff, and their temporal trends were found to differ among macronutrients and ecosystems,^15−17^ but the processes and factors controlling these variations have rarely been explored mechanistically. Molecular-level speciation of macronutrients in smoke and fire ash is key to predicting their geochemical behavior and cycling mechanisms (e.g., aqueous dissolution, pathways of entering environmental matrices), but it has rarely been characterized and remains poorly understood. For example, recent studies focused on pyrogenic carbon and contaminants associated with fire ash, with less efforts on the macronutrients.^18^

Our main research questions include: (1) what is the speciation of macronutrients in smoke and solid ash and what controls the speciation, (2) how does macronutrient speciation vary across ecosystems, and (3) how does speciation affect the postfire fate and transport of macronutrients? Filling these knowledge gaps is fundamental for evaluating the effects of fire on macronutrient cycling and the different impacts among ecosystems. Regarding questions 1 and #2, little is known about the macronutrient speciation and its variation in fire ash and smoke, as well as the governing factors. Although previous studies have characterized the mineralogy of biomass ash by X-ray diffraction,^12,14,19,20^ quantitative speciation of each macronutrient and variation among fire burning products of different ecosystems are very scarce. Only a few recent studies have characterized the speciation of P in ash from a particular fire, using nuclear magnetic resonance (NMR) spectroscopy and sequential extraction.^21,22^ Regarding smoke from wildland fires, although extensive studies have characterized its chemical composition,^23,24^ the speciation of macronutrients and the effects of fuel sources and fire conditions remain largely unexplored. With respect to question #3, previous studies have observed variation in the aqueous solubility and mobility of macronutrients among fire ash, while the causes were not clear.^12,25^

To answer these questions, this study characterized the chemical speciation of macronutrients in ash from wildland fires in a range of ecosystems as well as smoke and ash from simulated burns in the lab. Built on our recent studies that evaluated the interplay between biomass composition and fire thermal condition in controlling P and Ca speciation in biomass ash, leveraging laboratory controlled burning of uniform plant biomass,^26,27^ this study characterized and compared ash and smoke from the natural environments and more realistic burning. The chemical speciation of these macronutrients was related to their aqueous dissolution behavior. We focus on four mineral macronutrients (i.e., Ca, P, K, and Mg) because they are essential nutrients and the recycling of their aboveground biomass pools is a main source to soils and plant uptake.^4,28^ We hypothesize that chemical speciation of these macronutrients in fire ash and smoke differs between ecosystems, depending primarily on the elemental stoichiometry of fuel biomass and fire thermal conditions. To test this hypothesis, we comparatively characterized wildland fire ash from several representative ecosystems that are widely distributed across the world and differ in vegetation composition. In addition, ash and smoke from laboratory burning were also characterized, to identify the potential difference between the two fire burning products and evaluate the effect of burning severity. To obtain molecular-level quantitative speciation data, we used X-ray absorption spectroscopy (XAS), which is one of the most sensitive and informative tools for speciating elements in complex environmental samples^29^ and has rarely been used for characterizing wildland fire ash. To the best of our knowledge, this is the first study to characterize simultaneously the chemical speciation of the selected macronutrients and compare fire burning products (smoke and ash) from laboratory burning and wildland fires.

## 2 Materials and Methods

### 2.1 Sample Collection

Ash samples from two types of vegetation burning were collected for this study: (1) laboratory burning of biomass collected from Southeastern US Piedmont Forest (PD) and Coastal Plains (CP), and (2) experimental fires and wildfires in five ecosystems located in different regions around the world: a boreal forest (Canada), a temperate conifer forest (USA), a temperate eucalypt forest (Australia), two temperate heathland (UK and Spanish), and a subtropical savanna (South Africa) (Table 1). We deliberately selected these ecosystems because they differed greatly in vegetation types and elemental stoichiometry of biomass composition that is a major control of elemental speciation of fire ash.^27^

**Table 1:** Ecosystem Features and Elemental Compositions of Wildland Fire Ash Samples Compared in this Work

label	fire location	ecosystem	fire type and severity	rain before sampling	pH	TC (%)	TN (%)	elemental content (g kg^–1^ ash)				molar ratio	
								Ca	K	Mg	P	Ca/P	Ca/K
FORCAN	Canada	boreal forest	experimental (high)	N	8.0	53.5	1.1	31.5	3.0	4.2	1.0	24.4	10.2
MUSA-L	USA	temperate conifer forest	wildfire (low)	Y (5 mm)	9.0	7.2	0.4	48.1	8.0	8.4	2.5	14.9	5.9
WAUS	Australia	temperate eucalypt forest	experimental (high)	N	10.6	15.5	0.3	10.4	1.7	1.8	0.3	26.9	6.0
SPAU	Spain	temperate heathland	wildfire (high)	N	8.7	33.0	1.2	19.8	20.4	5.4	1.8	8.5	0.9
UKMA-H	United Kingdom	temperate heathland	wildfire (high)	Y (16 mm)	6.6	48.8	2.9	10.5	5.0	3.0	2.6	3.1	2.0
SASB	South Africa	subtropical savanna	experimental (low)	N	8.2	14.5	0.4	22.6	47.6	10.8	10.6	1.7	0.5

Details of the laboratory burning and sample collection can be found in Text S1. For the experimental fires and wildfires samples, a detailed description of the ecosystems, fire characteristics, and sampling strategies can be found in our previous study.^14^ We would like to note that, the ash samples of each ecosystem were sampled using a transect or composite sampling strategy, during which ash from multiple sites were pooled together.^14^ The sampling strategy homogenized a certain degree of ecosystem spatial heterogeneity in biomass composition and fire conditions. Samples from laboratory burning were included because laboratory burning enabled the simultaneous collection of both smoke and ash and the adjustment of fuel biomass moisture content (to mimic the typical conditions of prescribed fire and wildfire) that can hardly be achieved during wildland fires. All ash samples were ground and passed through sieve no. 35 (pore size = 0.5 mm). The homogenized samples were used in all analyses below.

### 2.2 Characterization of Elemental Composition

Elemental compositions (C, H, N, and S) of the fire ash samples were determined using a CHNS elemental analyzer (2400 Series II, PerkinElmer, Inc., Waltham, MA, USA). Approximately 1–10 mg of the homogenized samples was used, and the analysis was performed in triplicate. The total contents of other major macronutrients in fire ash, including K, Ca, Mg, and P, were analyzed by inductively coupled plasma optical emission spectrometry (ICP-OES). Specifically, 10 mg of fire ash was further heated at 550 °C for 3 h in a furnace prior to acid digestion, to completely mineralize the fire ash for total extraction (because fire ash consists of char that may not be extractable by acids). It is worth noting that this step may induce losses that affect measurement accuracy. The ash was digested with 1 mL of aqua regia (HCl/HNO_3_ = 3:1). After digestion, the sample was further diluted with 2% HNO_3_ (ytterbium and scandium were added as internal standards) prior to instrumental analysis. Composition of smoke on the filter was not analyzed due to sample form and limited quantity.

### 2.3 Synchrotron X-ray Absorption Spectroscopy

Synchrotron XAS data (K-edge X-ray absorption near-edge structure, XANES) were collected for P, K, and Ca in fire ash samples. The data were collected at the Tender Energy X-ray Absorption Spectroscopy (TES) beamline at the National Synchrotron Light Source II at the Brookhaven National Laboratory (Upton, NY). The pulverized fire ash samples were spread on Kapton tape on a multisample holder, which was placed in a helium-flowed chamber for XANES spectra collection. The data were collected in the fluorescence mode using a four-element Si(Li) drift detector, and the beam size (unfocused) was 2 mm × 5 μm. The monochromator was first calibrated with gypsum prior to sample spectrum collection.

Spectra for P, K, and Ca were collected, respectively, between 2100–2250 eV, 3570–3710 eV, and 4015–4200 eV, with varying step sizes. The P and Ca K-edge spectra of hydroxyapatite were collected along with each batch of samples for postcollection energy calibration. The P K-edge spectrum was calibrated by setting the first derivative peak at 2152.6 eV and the Ca K-edge spectrum by calibrating the pre-edge peak of apatite at 4040 eV.^29^ Two scans were collected for each sample. The duplicated spectra were merged, background subtracted and normalized, before linear combination fitting (LCF), all of which were performed using the Athena package.^30^ Additional details of the spectral collection and LCF analysis can be found in Text S2.

### 2.4 Sequential Fractionation of Macronutrients

Sequential fractionation of P and the other three macronutrients in fire ash was conducted following the modified Hedley sequential extraction method.^31^ Although the Hedley sequential extraction was originally used to fractionate P into different chemical pools, the partitioning of other macronutrients (Ca, K, and Mg) into the pools may help explore their speciation and thus was also analyzed. Specifically, 200 mg of the pulverized fire ash was added to a 50 mL polypropylene centrifuge tube and sequentially extracted using 40 mL of the extraction solution. The sample tubes were continuously shaken at 120 rpm and room temperature. The samples were first extracted using deionized water, followed by 0.5 M NaHCO_3_, 0.1 M NaOH, and 1 M HCl, each lasting 16 h. At the end of each step, the tubes were centrifuged at 3750 rpm for 20 min to separate the liquid and solid. The concentrations of P, Ca, K, and Mg in the extracted solutions from each step were determined using ICP-OES.

### 2.5 Aqueous Dissolution Experiments

Aqueous solubility of the macronutrients in ash reflects their postfire mobility (often driven by precipitation) and thus was determined and related to their speciation. Kinetics of aqueous dissolution of macronutrients were measured under two conditions: (1) no pH adjustment using deionized water, which resulted in a pH ranging from 6.6 to 11.5 and (2) pH = 6.0 using 50 mM acetate buffer. The fire ash was mixed with solutions at a solid/liquid ratio of 200 mg/40 mL of solution. The solution was constantly stirred with a magnet bar. The concentration of macronutrients in the aqueous phase was closely monitored over a 30 h period, with 1 mL samples collected at 0.5, 1, 2, 5, 8, 12, and 30 h after mixing. The solution was filtered (0.45 μm) and diluted with 2% nitric acid prior to analysis of Ca, Mg, K, and P concentrations using ICP-OES. The solution pH was temporally measured and remained stable during the experiment.

Several dissolution kinetics models (including several commonly used first- and second-order models) were used to fit the nutrient release data and among them the Korsmeyer–Peppas model showed the best fit.^32^ Therefore, it was used to derive the kinetic parameters that were used to compare the release rates among the macronutrients and between pHs. The general form of the equation for this model is
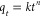
where *q*_*t*_ is the concentration of nutrients released at time *t*, *k* is the kinetic rate constant incorporating the structure and geometry of fire ash, and *n* is the release exponent, indicative of the nutrient release mechanism.

### 2.6 Macronutrient Loadings from Fire

The total and readily available (extracted by 0.5 M sodium bicarbonate solution) loads of macronutrients present in the fire ash provide insights into the immediate fire-induced changes to their aboveground pools. They were calculated for two ecosystems with different fire and vegetation characteristics: temperate eucalypt forests and savanna. The two loads were calculated from the macronutrient chemistry (total nutrient content and readily available %) determined in this study, as well as fire ash load (kg/ha) from previous studies (Tables S6 and S7) measuring ash loads directly in the field. The calculation was based on the following two equations:
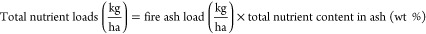

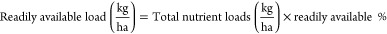
where total nutrient content in ash and readily available nutrient (%) were measured in this study. Readily available nutrients are the combined H_2_O and NaHCO_3_ pools from sequential extraction study. To compare the difference in individual macronutrient load between two ecosystems, a one-way analysis of variance was conducted. The significance of differences between treatments was assessed using Student’s *t*-test, with a threshold for significance set at *P* < 0.05. Statistically significant differences among treatments were indicated by assigning different letters. All statistical analyses were performed using JMP software (SAS Institute, Cary, NC).

## 3 Results and Discussion

### 3.1 Physicochemical Properties of Wildland Fire Ash

Elemental contents and stoichiometries of the wildland fire ash were characterized and differed broadly (Table 1). The total C content (including inorganic and organic C) ranging from ∼7.2 to 53% by weight and content ranges of the four macronutrients were Ca—10.4 to 48.1 g/kg; K—1.7 to 48 g/kg; Mg—1.8 to 10.8 g/kg; and P—0.3 to 10.6 g/kg. The different contents resulted in a difference in elemental stoichiometry, with Ca/P and Ca/K molar ratios varying between 1.7 to 27 and 0.5 to 10.2, respectively (Table 1). The total C content reflects a difference in combustion completeness because organic C is preferentially volatilized during burning and total C generally decreases as burning becomes more completed (inorganic C portion is relatively small),^13^ while ash produced from incomplete combustion shows a higher concentration of charred organic.^33^ For example, fire ash from boreal forest and UK heathland has a relatively high TC of 53.5 and 48.8%, respectively, indicating a low burning completeness.

Difference in macronutrient contents and stoichiometry is most likely a collective result of varying composition in fuel biomass and combustion completeness because these elements have relatively high volatilization temperatures and tend to remain in the solid ash.^9^ For example, P content varied between 0.3 g/kg (temperate eucalypt forest) and 10 g/kg (subtropical savanna) and Ca content ranged between 10 g/kg (temperate eucalypt forest) and 48 g/kg (temperate conifer). The high content of a specific nutrient is most likely due to a high content in the biomass and/or high degree of combustion completeness. In comparison, fire ash from subtropical savanna has relatively small Ca/P and Ca/K molar ratios (Ca/P = 1.7 and Ca/K = 0.5). Fire ash from woody ecosystems, such as boreal and temperate forests and temperate eucalyptus forest, have relatively high Ca contents and large Ca/P and Ca/K molar ratios (Ca/P > 14 and Ca/K > 6). The observed difference in macronutrient stoichiometry is thus primarily caused by variation in ecosystem vegetation composition (and their macronutrient composition),^34^ although may also be contributed by different behaviors of the macronutrients during fires (e.g., differential volatilization temperature).^11^ In general, mineral macronutrient content in grass biomass follows an order of K > Ca ≅ Mg > P,^35,36^ while that in wood biomass (particularly stem and bark) follows an order of Ca > K > Mg ≅ P.^37,38^ Therefore, fire ash from grass-dominated ecosystems generally showed relatively small Ca/P and Ca/K molar ratios compared to those from forest ecosystems. The elemental stoichiometry affects macronutrient speciation in fire ash and ultimately nutrient loads from fires, as demonstrated below.

### 3.2 Chemical Speciation of Macronutrients in Wildland Fire Ash

The chemical speciation of three abundant and closely associated macronutrients, P, Ca, and K, was characterized by their K-edge XANES analysis and sequential extraction, and the results were discussed separately.

**Figure 1:**
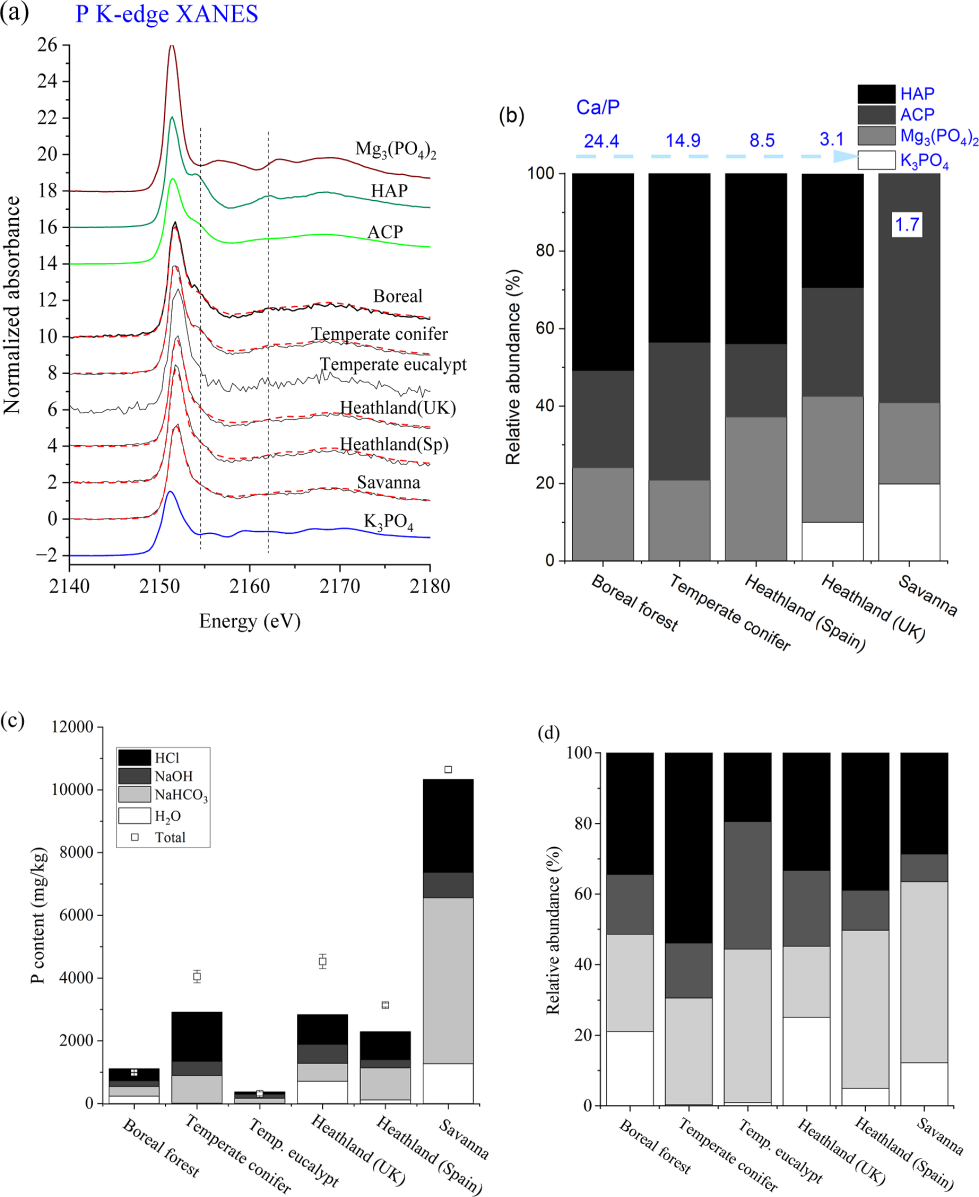
(a) Phosphorus K-edge XANES spectra of wildland fire ash. Reference spectra were from our previous study.^26^ (b) Relative abundance of different P species in fire ash samples as quantified by LCF of their P XANES spectra, Ca/P ratios were also included. Distribution of P in the Hedley fractionation pools, expressed as the mass concentration (c) and percentage (d).

Speciation estimated by LCF revealed that P existed dominantly as Ca phosphates (others being Mg and K phosphates), although the relative abundance varied among ash samples (Figure 1). Calcium phosphates (>75% of total P, with ∼50% being apatite) were the most abundant in fire ash of boreal and temperate coniferous forests, with the rest being Mg phosphate (Figure 1b). Less Ca phosphates existed in fire ash from heathland than from boreal and temperature coniferous forests, and subtropical savanna fire ash has the least Ca phosphates, with no crystalline hydroxyapatite identified (Figure 1b). The result was consistent with our previous result that large Ca/P ratios in biomass tend to induce more Ca phosphates and the Ca phosphates become more crystalline at elevated temperature.^26,39^ In general, sequential extraction data agreed with the speciation and chemical properties of the fire ash. The proportion of the water extractable fraction was related to the aqueous solubility of phosphate minerals that often depends on solution pH. For example, the fire ash from UK heathland, boreal forest, and savanna possessed a relatively low pH (6.6, 8.0, and 8.2, respectively) among all the tested samples, thus having relatively high H_2_O–P of ∼25%, 21%, and 10%, respectively (Figure 1c,d). The HCl extractable P corresponded to insoluble P minerals, primarily the abundant crystalline apatite identified by XANES fitting.^26^ The bicarbonate extractable P was likely soluble phosphates and phosphates associated with carbonates. NaOH-extractable P accounted for 10 to 20% (except for eucalypt that has about 30%), suggesting the presence of mineral-adsorbed P, such as those adsorbed by iron, aluminum, and clay minerals. These minor species can hardly be identified and quantified by LCF of P K-edge XANES because it is not sensitive to species without strong spectral feature and has a large fitting uncertainty.^40^

**Figure 2:**
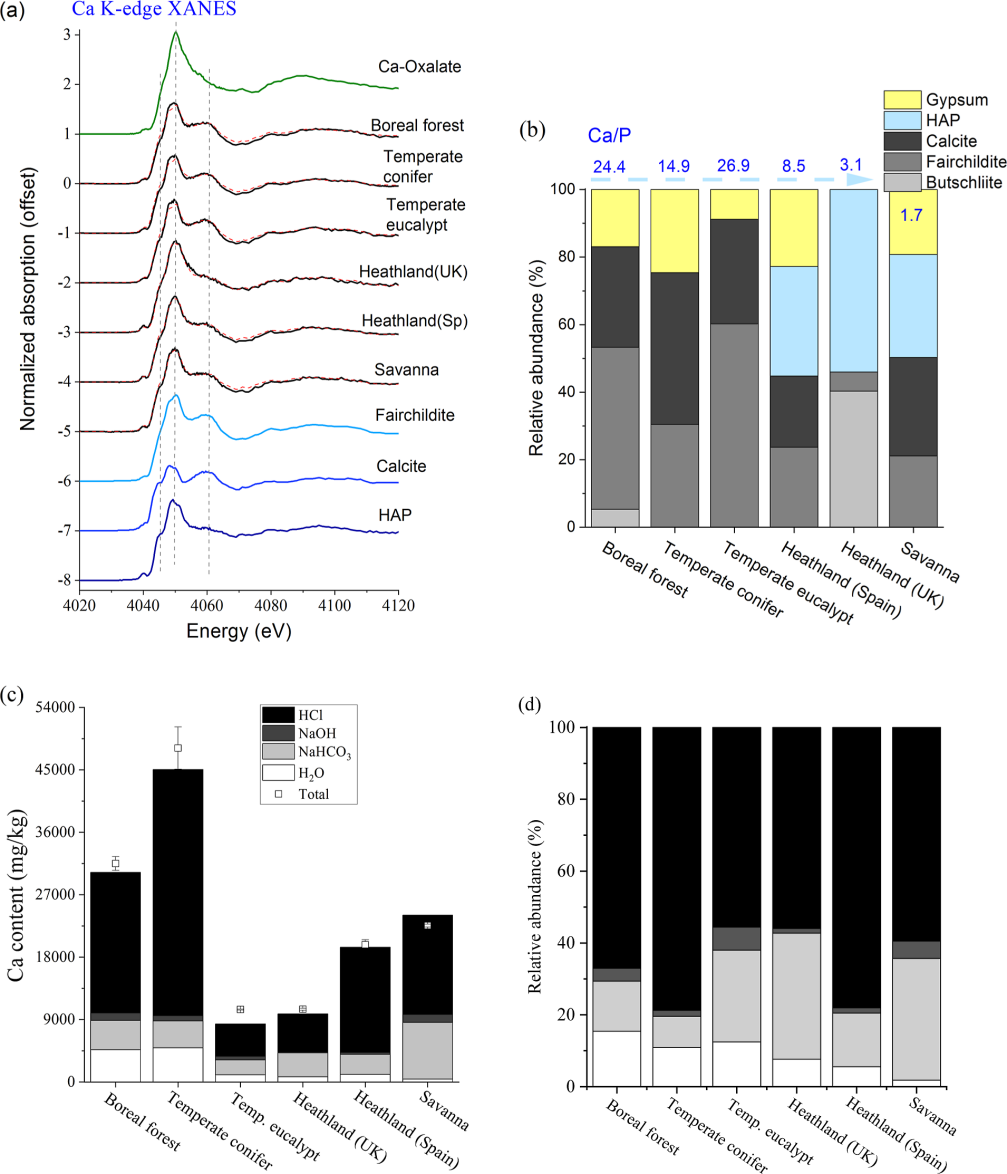
(a) Calcium K-edge XANES spectra of wildland fire ash samples collected from various sites. Reference spectra were from our previous study.^27^ (b) Relative abundance of Ca species in fire ash as quantified by LCF of their Ca XANES spectra. (c) Distribution of Ca in the Hedley fractionation pools, expressed as the mass content (c) and percentage (d).

The Ca K-edge XANES spectra of the ash samples of the three ecosystems with large Ca/P molar ratios (>10) resembled those of Ca carbonates (the slight left shift of the white line at 4050 eV and absorption at 4060 eV), specifically butschliite, fairchildite, and calcite (Figure 2a). In comparison, XANES spectra of heathland and savanna ash were similar to that of hydroxyapatite (4040, 4045, and 4050 eV). These Ca minerals were among the common Ca species identified in plant ash by XRD.^19,20^ Speciation quantified by LCF showed the predominant abundance of fairchildite and calcite (total 75–91%) in ash from the three forest ecosystems, with the rest being Ca sulfate (Figure 2b). In comparison, ash samples from heathland and savanna consisted of abundant hydroxyapatite (30–54%), in addition to the carbonates and sulfate. The results that Ca/P controls the abundance of apatite as a Ca species agreed with our recent findings based on laboratory burning of uniform biomass with different Ca/P molar ratios^27^ and the stoichiometric abundance of Ca in woody biomass.^37,38^ The relative abundance of butschliite, fairchildite, and calcite are possibly dependent on fire thermal condition and biomass composition (e.g., Ca/K ratio), based on the following arguments. Three Ca–K double carbonates [butschliite, fairchildite, and K_2_Ca_2_(CO_3_)_3_] were known to experience phase transition as temperature increases, with butschliite to fairchildite at about 547 °C.^41^ The UK heathland sample consisted of ∼40% butschliite and 5.7% fairchildite, suggesting a relatively low burning temperature compared with that of Spain heathland (which has 23.7% fairchildite and 21.1% calcite). Regarding the sequential fractionation, about 60 to 80% of Ca was soluble in 1 M HCl, with the rest being soluble in water or 0.5 M NaHCO_3_ (Figure 2c,d). The partitioning among the extraction pools corresponds to the Ca speciation results that apatite, carbonates, and gypsum were the dominant species, whose aqueous solubility is regulated by pH.

**Figure 3:**
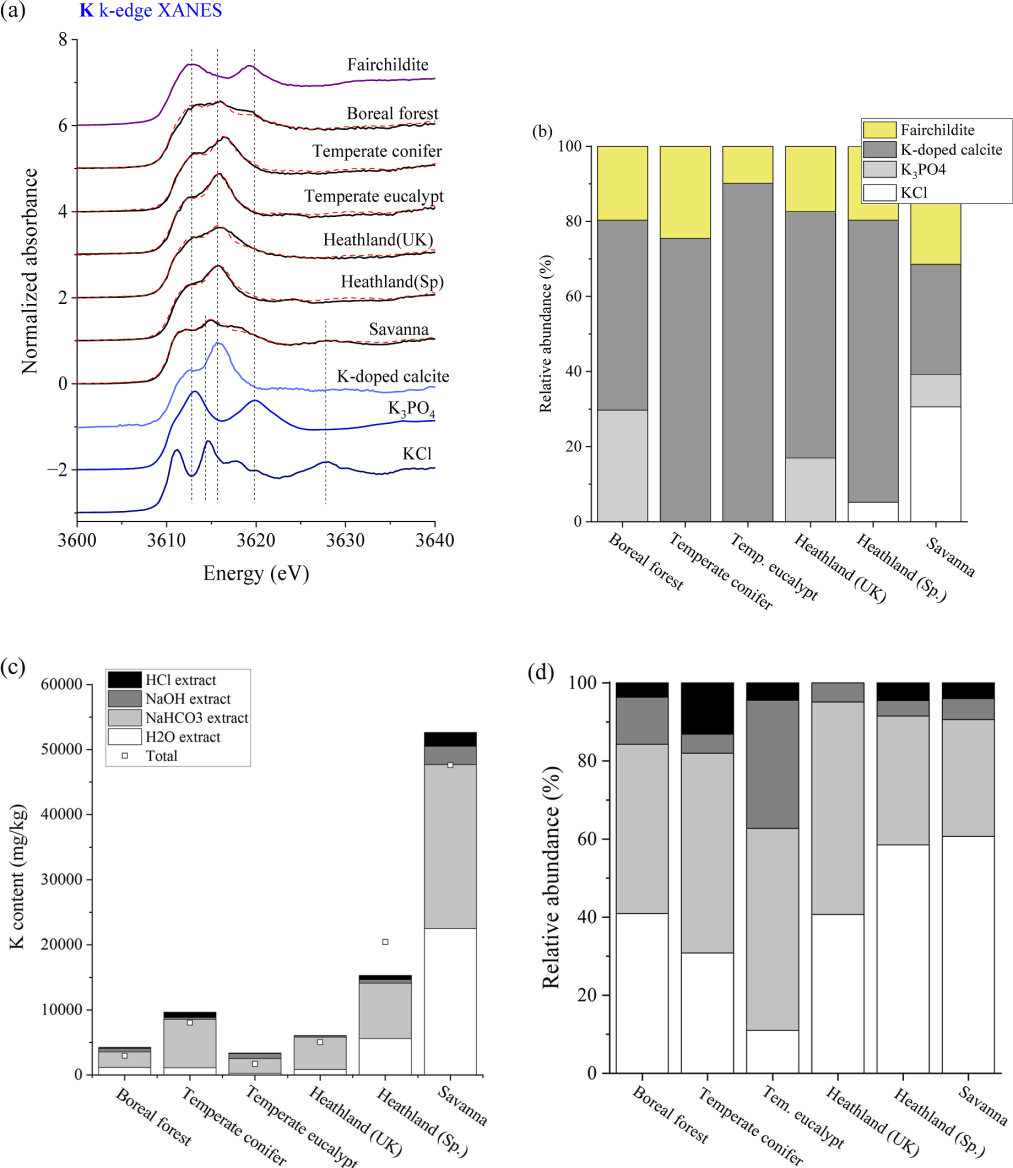
(a) Potassium K-edge XANES spectra of wildland fire ash. (b) Relative abundance of K species in fire ash as quantified by LCF of their K XANES spectra. Distribution of K in the Hedley fractionation pools, expressed as the mass concentration (c) and percentage (%) (d).

Analysis of the K K-edge XANES data showed K-doped calcite and fairchildite being the dominant K species, which in total accounted for 56% to 100% (Figure 3a,b). Two other species, KCl and K_3_PO_4_, were also identified in some of the fire ash, with KCl identified in savanna and Spain heathland (30% and 5%, respectively) and K_3_PO_4_ in boreal forest, savanna, and heathland (UK). The predominance of carbonates and phosphate aligns with the lithophile nature of K. The speciation results correspond to the relatively high solubility of K that most K (>80%) partitioned in water and NaHCO_3_ pools (Figure 3c,d).

### 3.3 Effects of Burning Severity on Macronutrient Speciation of Fire Ash and Smoke

**Figure 4:**
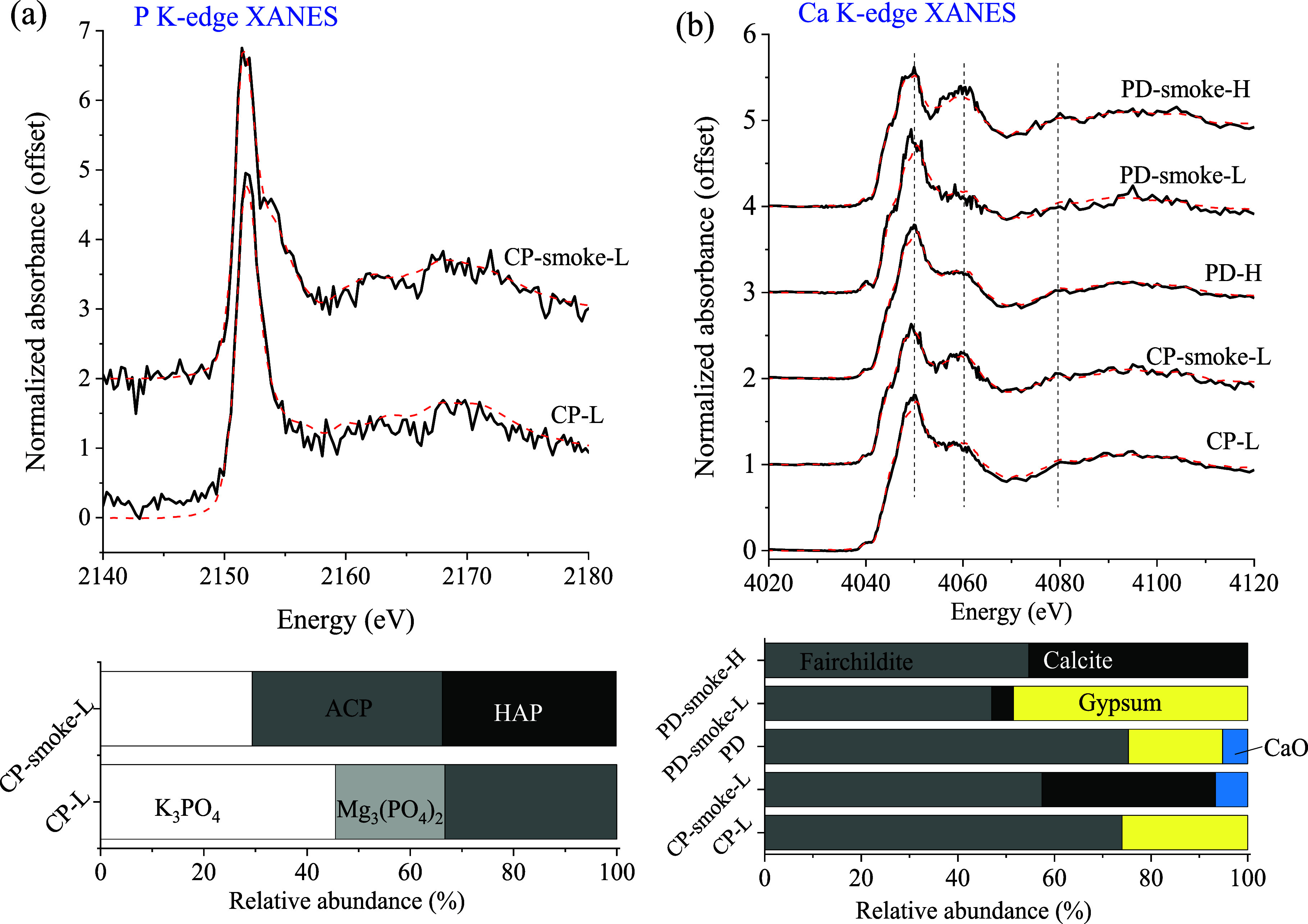
K-edge XANES spectra and speciation of bottom ash and smoke from the simulated burning for P (a) and Ca (b). CP and CP-smoke are bottom ash and smoke (filter) from laboratory burning of biomass from the CP, respectively. PD-H and PD-smoke-L (H) are bottom ash and smoke (filter) of Piedmont Forest burning (low and high severity), respectively.

For the laboratory burns, the overall P and Ca species identified in both ash and smoke samples agreed with those in wildland fire ash samples. Nevertheless, speciation differences were observed between: (1) ash and smoke from the same burn and (2) ash or smoke from two different burns (Figure 4). Phosphorus speciation data showed that more Ca phosphates (especially crystalline hydroxyapatite) were found in the smoke than in the ash from the burning of CP biomass at 10% moisture (low severity) (Figure 4a). Regarding Ca speciation, the smoke of CP burning contained relatively abundant calcite (∼35%) and Ca oxide (8%), in addition to fairchildite (Figure 4b). In comparison, the corresponding ash contained mainly fairchildite (74%) and gypsum. The speciation in the ash and smoke samples from the burning of PD biomass follows similar trends, with the ash containing mainly fairchildite (75%) and gypsum (20%) (Figure 4b). Differences were also observed between PD smoke samples produced under two moisture levels, with smoke from 4% moisture (high burning severity) containing ∼45% calcite and smoke from 10% moisture (low burning severity) containing gypsum (50%) and minor calcite (5%), in addition to the dominant fairchildite (∼50%).

Difference in chemical speciation between smoke and ash, specifically the presence of more crystalline Ca phosphates and calcite in smoke compared to ash, could be a collective result of many physical and chemical processes occurred during fire burning. First, smoke particles transported to the atmosphere may experience more thermal intensity (i.e., higher temperature) than that of ash because the smoke particles are generally heated by fire frame, whose temperature is higher than that on the ground.^42^ This may contribute to the presence of more crystalline apatite and calcite in smoke than in ash, because their abundance increases with temperatures.^43^ Second, the difference may also be caused by chemical and mass fractionation during a fire. For example, macronutrients are known to be differently transferred^11^ and small and light particulates (whose composition are size-dependent) are preferentially transferred into the atmosphere,^23,44^ contributing to the speciation difference between smoke and ash.

### 3.4 Aqueous Solubilization of Macronutrients in Fire Ash

**Figure 5:**
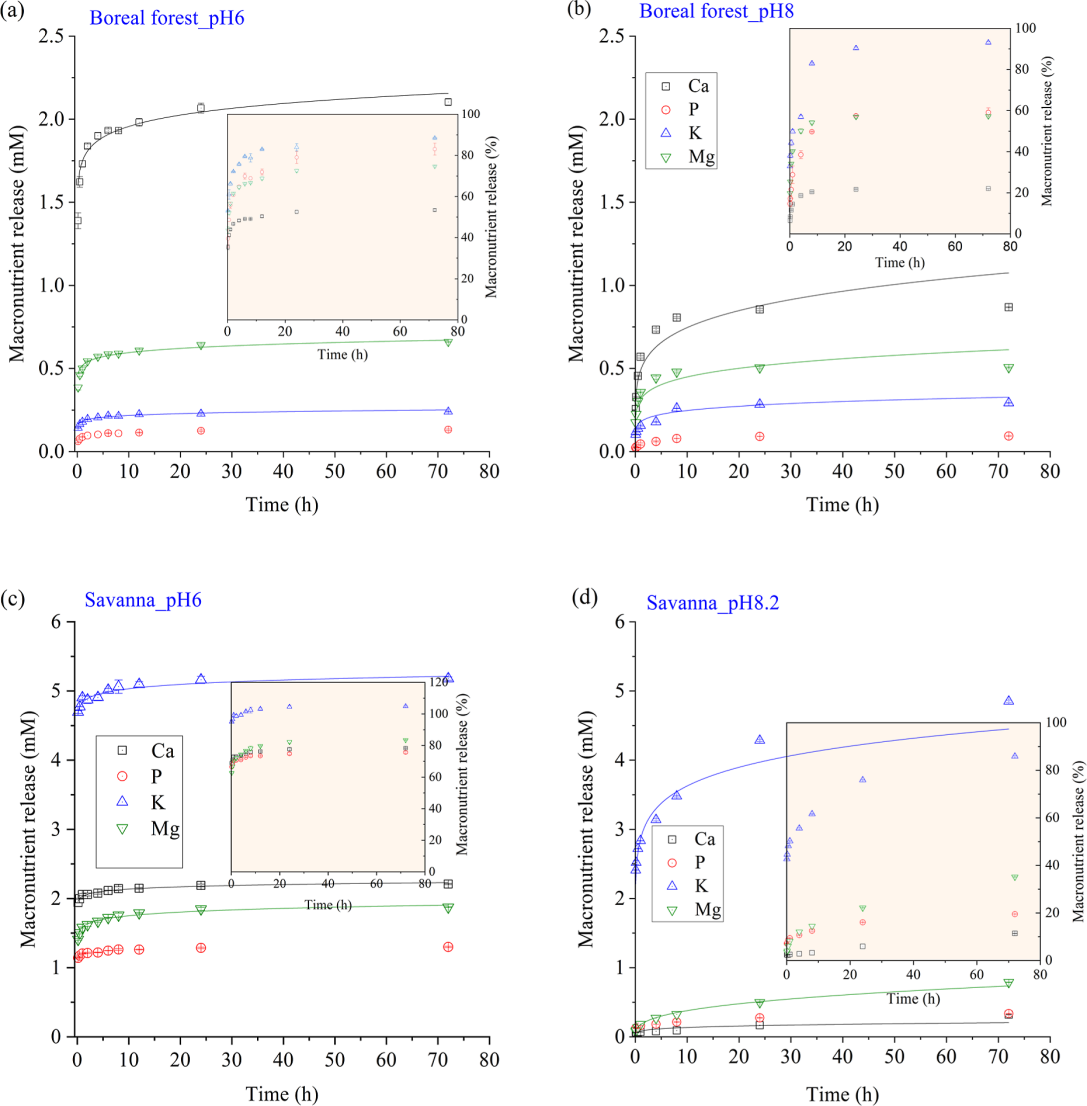
Release kinetics of Ca, K, Mg, and P for the boreal forest sample at a buffered pH = 6.0 (a) and unadjusted pH = 8.0 (b) and for the Savanna sample at pH = 6.0 (c) and unadjusted pH = 8.2 (d). The solid lines were curve fitting with the Korsmeyer–Peppas model. Error bar is standard deviation of triplicate experiments (*n* = 3).

The solubilization of macronutrients (except for K) was dependent on pH and the released concentration (or % release) increased as pH decreased from the alkaline pH to 6.0 (Figures 5 and S3). The aqueous solubilization behaviors agreed with their speciation, with Ca and P existing primarily as carbonates and phosphates whose solubilities depend on pH.

Regarding the kinetics, at a buffered pH = 6.0, all four macronutrients were rapidly released within the first 5 h and then stabilized with no significant release beyond that. In comparison, dissolution kinetics at the intrinsic pH (most were alkaline) were relatively slow. Kinetics model fitting showed variations in the rate constant *k* among the samples for the same macronutrients and between the two pH conditions (Table S5). For the same macronutrient, the constant *k* was overall larger for samples with higher nutrient contents and at a pH = 6 compared to the alkaline conditions. For example, the *k* values for Ca release or K release correlated linearly with their total contents in ash (Figure S4). It is likely that higher macronutrient contents correspond to more surface exposures and thus larger *k* values. The larger *k* values at lower pH correspond to more soluble species, as compared to that at higher pHs.

The release exponent *n*, which indicates the release scheme, ranged from 0.017 to 0.087 for samples at a pH = 6 and from 0.06 to 0.34 for samples without pH adjustment (mostly alkaline condition). Decrease of the release exponent *n* (from 1 to 0) corresponds to the transition from reaction-controlled to mass transfer (diffusion)-controlled release.^45^ The relatively small *n* at low pH indicates primarily mass transfer limited release of macronutrients from the solids (mineral dissolution was fast and not the rate-limited step). The relatively large *n* at high pH indicates possibly reaction-controlled release (e.g., dissolution-controlled).^46^ The exact mechanisms are still unclear and need further study.

### 3.5 Thermochemistry of Macronutrients during Vegetation Fires and the Effects on Their Postfire Cycling in Soils

Results from this study advance our understanding of the thermochemistry of macronutrients during fires and its variation across ecosystems. This can help elucidate the impact of fire disturbances on ecosystem macronutrient budget and cycling.

This study, for the first time, used synchrotron XAS to simultaneously speciate P, Ca, and K, which generated quantitative speciation of the studied macronutrients for ash and smoke from several widely distributed ecosystems and lab burning. For example, the chemical species of these macronutrients and their relative abundance were determined (Figures 1–3). The quantitative speciation data, along with the diverse sample sets we selected (wildland fire ash and smoke and ash from laboratory burning) revealed that macronutrient speciation varies across ecosystems and is affected by fuel biomass composition (specifically macronutrient stoichiometry) and fire thermal conditions. For example, Ca phosphates (the dominant P species) are more abundant in forest fire ash than in fire ash from savanna and heathland because of relatively large Ca/P molar ratios of the woody fuel^34^ and relatively small Ca/P molar ratios of grass fuels.^36,47^ This result aligns with our previous observations from controlled burning of plant compartments (e.g., leaf, fruit, twig, and wood), which showed the formation of abundant apatite in ash of plant compartments with large Ca/P molar ratios.^26^ Regarding the speciation of Ca, this study revealed that although Ca (existed primarily as organic complexes in biomass^48^) was similarly transformed into double carbonates and carbonates in ash and smoke, phases and relative abundance of the minerals could be affected by fire temperature, based on thermal stability of the minerals.^27,41^

Second, the detailed macronutrient speciation and aqueous dissolution data obtained in this study help predict the effects of fire ash in the postfire environment. In the absence of fire, microbes primarily mediate the mineralization of organic matter and liberate mineral macronutrients into soils.^29,49^ With the mineralization of organic matter by fires and the transformation of nutrient speciation, it is important to understand the mobility and transport mechanisms of macronutrients in fire ash. This study revealed the aqueous dissolution behaviors of macronutrients that agree with their chemical speciation in ash (Figure 5), which is an advancement to previous studies that primarily reported phenomenological observations of water solubility of macronutrients in fire ash.^13,25,50^ For example, the different solubilities among Ca, P, and K and the pH dependencies of Ca and P can be reconciled by their chemical speciation.

Fire-induced mineralization of macronutrients and its variation are likely to have far-reaching effects on postfire cycling of the macronutrients. First, the pathways and rate of macronutrient returning to soils will change after fires, from microbe-mediated processes to most likely mineral weathering processes that depend on precipitation and soil chemical properties, such as pH (Figure 5). Second, variations in speciation of the same macronutrient among ecosystems (as a result of varying elemental stoichiometries and severities) may also affect the pathway and rate of macronutrient mobilization across ecosystems. For example, aqueous solubility of P is affected by the abundance of apatite in fire ash and burning completeness of the ash, which is regulated by the Ca/P molar ratio and fire thermal conditions.^26,51^ Third, our results also showed varying speciation and aqueous solubilities of different macronutrients in fire ash, which will affect their postfire mobilization and availability in soils.

**Figure 6:**
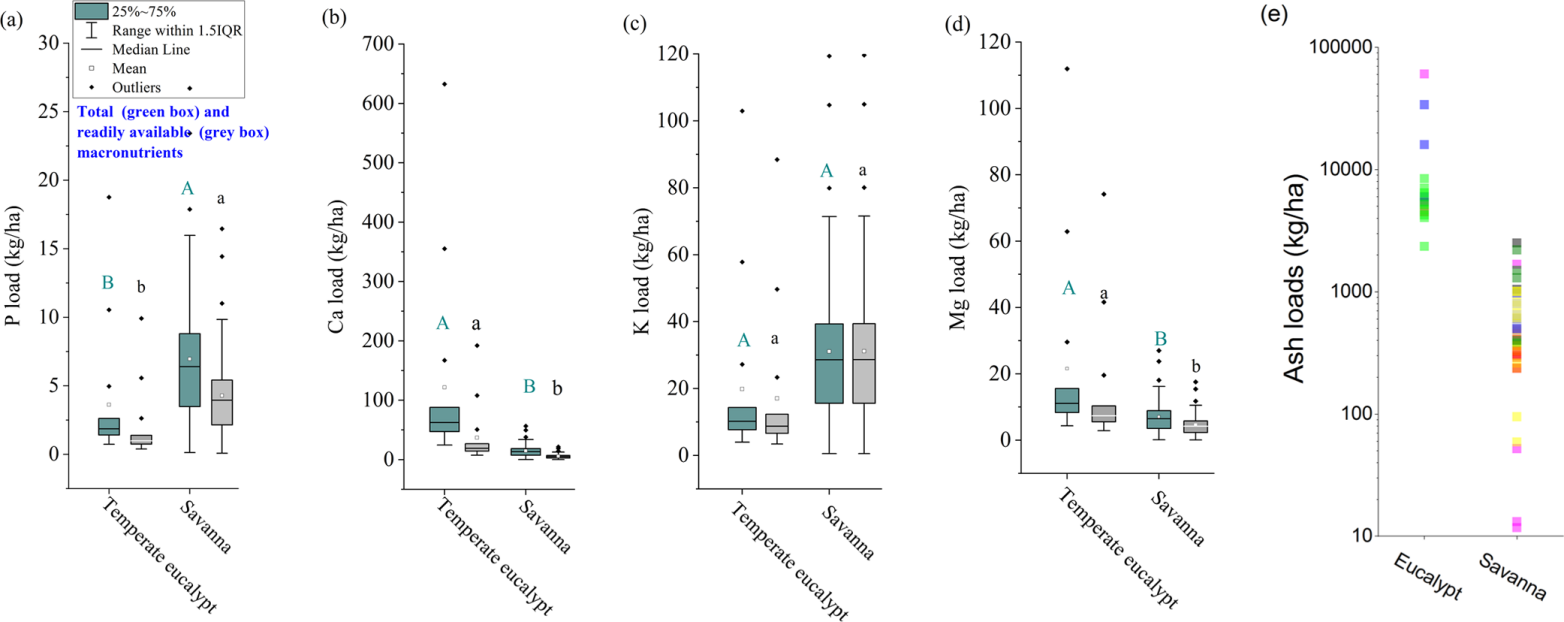
Total and readily available (H_2_O + NaHCO_3_ extractable pools) macronutrients from fire ash for two representative ecosystems—temperate eucalypt and savanna. Panel (a) to (d) were for P, Ca, K, and Mg, respectively. The ash load data from previous studies were also plotted, with different sources labeled with different colors (e). Student’s *t*-test was performed to compare the difference in individual macronutrient load between two ecosystems. Different letters were assigned when the difference was significant (*p* < 0.005). Capital letters represent comparison between total loads, while lowercase letters represent comparison between readily available loads.

To demonstrate the differential effects of fires on ecosystems and the upscaled (soil profile to ecosystem scale) effects of macronutrient chemistry (i.e., content, speciation, and aqueous solubility) in fire ash on postfire nutrient cycling, we calculated and compared macronutrient loading and availability for two ecosystems, combining the chemical data from this study with ecosystem-dependent ash loading data (Figure 6). The result showed that the loads of individual macronutrient differed between ecosystems, as a collective result of different fire ash loads and ash chemical composition.^52^ Ash amount and composition are determined by several factors, the two key ones being fuel properties (amount, physical and chemical characteristics) and fire behavior (which determines fuel consumption).^9^ For example, fires in eucalypt forests generally produce higher ash loads than savanna fires (Tables S6 and S7), the loads of some macronutrients (such as P and K) can be the opposite (Figure 6) because of relatively high nutrient contents in the savannah fire ash. Mineral macronutrient content in grass biomass follows an order of K > Ca ≅ Mg > P (e.g., K:Ca:Mg:P molar ratio is 21.6–10–8.0–6.1),^35,36^ leading to relatively high loads of K than Ca from fires in savanna. In contrast, the load of Ca is much higher than that of K in eucalypt forests, as a result of the stoichiometric abundance of Ca in wood biomass.^34,37^ The different loads and aqueous solubility of the studied macronutrients will condition the flux of these macronutrients returning to soils after fires or moving through the landscape via water or wind erosion. For example, the relatively large load and solubility of K found in savanna ash indicate that this type of fire induces large and rapid fluxes of K (available loads), while Ca may be moving at large quantities in fire-affected forests (Figure 6).

The above analysis demonstrates the importance of ash macronutrient chemistry and its variations across ecosystems in quantifying the immediate disturbance of fires to aboveground macronutrient pools (the quantity and quality of macronutrients). With the complexity and heterogeneity of ecosystem and fire conditions, it is worth noting potential uncertainties of the results and interpretation. First, our analysis of a homogenized ash sample could not reflect the spatial heterogeneity in the fuel biomass composition and fire burning conditions, which are the norm in the real world. Second, the calculation of total and readily available loads of macronutrients involved two measurements, ash loading and ash chemistry (nutrient content and % of readily soluble nutrient), each of which can be variable. Herein, only the variation in ash loading was accounted for. Third, the total and readily available loads of macronutrients calculated herein represent only the potential pool size of fire-derived macronutrients but not the actual amounts that may return to soils. Because fire ash is commonly subjected to various postfire physical processes, such as winds and surface runoff, which remove fire ash from the burned sites and affect the fluxes of ash-derived macronutrient entering soils.^53,54^

## 4 Environmental Implications

This study performed comprehensive chemical analyses of macronutrients and generated a large quantitative data set of macronutrient geochemistry in fire ash, which helped gain a mechanistic understanding of how fires differentially transform macronutrients, in terms of their chemical speciation and aqueous solubility. Considering the effects of fires on the burned ecosystems and surrounding water bodies and atmosphere, such data set and improved understanding can help study relevant ecosystem services and processes that are disturbed by fires.

Results from this study will first contribute to the exploration of the postfire dynamics of ecosystem nutrient status because ash returning to soils represents the main sink of fire ash and can change soil nutrient content and availability that affect aboveground primary productivity. The speciation and aqueous solubility of macronutrients are fundamental for determining the pathways, rates, and chemical forms of macronutrient entering soils, ultimately their availability for plant uptakes.

In addition to the altered recycling to soils, fires also induce macronutrient transfer to the atmosphere and water bodies, through atmospheric emssion^55^ and erosion by wind and precipitation.^56^ Macronutrient chemistry of smoke and ash is important for determining the forms and fluxes of nutrient export from the burned environment and effects on the received environments, such as the fertilization of lake and ocean by smoke and eroded fire ash.^57,58^

Although differences in fire disturbances among ecosystems have long been recognized and studied, such as fire regimes, combustion behavior and emission, and changes to nutrient pools, there are knowledge gaps in connecting fire behaviors and postfire cycling of the mineral macronutrients for different ecosystems. Our multiscale analysis of the variations in macronutrient chemistry and loading among ecosystems underlines the need to account for such variations in evaluating fire disturbance to terrestrial ecosystems.

## Supporting Information Available

Information of laboratory burning; description of synchrotron data collection and analysis; speciation data of Ca, K, and P from LCF of the K-edge XANES spectra; temporal macronutrient release data and the kinetics fitting results; and raw data for calculation of the total and available loads of macronutrients (PDF)

## References

[ref1] Walker T. W.; Syers J. K. The fate of phosphorus during pedogenesis. Geoderma 1976, 15 (1), 1–19. 10.1016/0016-7061(76)90066-5.

[ref2] Cole D.; Rapp M. Elemental cycling in forest ecosystems. In Dynamic Properties of Forest Ecosystems; Cambridge University Press, 1981; Vol. 23, pp 341–409.

[ref3] Vesterdal L.; Clarke N.; Sigurdsson B. D.; Gundersen P. Do tree species influence soil carbon stocks in temperate and boreal forests?. For. Ecol. Manag. 2013, 309, 4–18. 10.1016/j.foreco.2013.01.017.

[ref4] Sohrt J.; Lang F.; Weiler M. Quantifying components of the phosphorus cycle in temperate forests. WIREs Water 2017, 4 (6), e1243 10.1002/wat2.1243.

[ref5] Pellegrini A. F. A.; Refsland T.; Averill C.; Terrer C.; Staver A. C.; Brockway D. G.; Caprio A.; Clatterbuck W.; Coetsee C.; Haywood J. D.; Hobbie S. E.; Hoffmann W. A.; Kush J.; Lewis T.; Moser W. K.; Overby S. T.; Patterson W. A.; Peay K. G.; Reich P. B.; Ryan C.; Sayer M. A. S.; Scharenbroch B. C.; Schoennagel T.; Smith G. R.; Stephan K.; Swanston C.; Turner M. G.; Varner J. M.; Jackson R. B. Decadal changes in fire frequencies shift tree communities and functional traits. Nat. Ecol. Evol. 2021, 5 (4), 504–512. 10.1038/s41559-021-01401-7. 33633371

[ref6] Johnson D.; Murphy J. D.; Walker R. F.; Glass D. W.; Miller W. W. Wildfire effects on forest carbon and nutrient budgets. Ecol. Eng. 2007, 31 (3), 183–192. 10.1016/j.ecoleng.2007.03.003.

[ref7] Gray D. M.; Dighton J. Mineralization of forest litter nutrients by heat and combustion. Soil Biol. Biochem. 2006, 38 (6), 1469–1477. 10.1016/j.soilbio.2005.11.003.

[ref8] Pausas J. G.; Bond W. J. On the three major recycling pathways in terrestrial ecosystems. Trends Ecol. Evol. 2020, 35 (9), 767–775. 10.1016/j.tree.2020.04.004. 32381268

[ref9] Bodí M. B.; Martin D. A.; Balfour V. N.; Santín C.; Doerr S. H.; Pereira P.; Cerdà A.; Mataix-Solera J. Wildland fire ash: production, composition and eco-hydro-geomorphic effects. Earth-Sci. Rev. 2014, 130, 103–127. 10.1016/j.earscirev.2013.12.007.

[ref10] Kauffman J. B.; Cummings D. L.; Ward D. E.; Babbitt R. Fire in the Brazilian Amazon: 1. Biomass, nutrient pools, and losses in slashed primary forests. Oecologia 1995, 104 (4), 397–408. 10.1007/BF00341336. 28307654

[ref11] Raison R. J.; Khanna P. K.; Woods P. V. Mechanisms of element transfer to the atmosphere during vegetation fires. Can. J. For. Res. 1985, 15 (1), 132–140. 10.1139/x85-022.

[ref12] Santín C.; Doerr S. H.; Otero X.´ L.; Chafer C. J. Quantity, composition and water contamination potential of ash produced under different wildfire severities. Environ. Res. 2015, 142, 297–308. 10.1016/j.envres.2015.06.041. 26186138

[ref13] Pereira P.; Úbeda X.; Martin D. A. Fire severity effects on ash chemical composition and water-extractable elements. Geoderma 2012, 191, 105–114. 10.1016/j.geoderma.2012.02.005.

[ref14] Sánchez-García C.; Santín C.; Neris J.; Sigmund G.; Otero X. L.; Manley J.; González-Rodríguez G.; Belcher C. M.; Cerdà A.; Marcotte A. L.; Murphy S. F.; Rhoades C. C.; Sheridan G.; Strydom T.; Robichaud P. R.; Doerr S. H. Chemical characteristics of wildfire ash across the globe and their environmental and socio-economic implications. Environ. Int. 2023, 178, 108065 10.1016/j.envint.2023.108065. 37562341

[ref15] Kong J. J.; Yang J.; Bai E. Long-term effects of wildfire on available soil nutrient composition and stoichiometry in a Chinese boreal forest. Sci. Total Environ. 2018, 642, 1353–1361. 10.1016/j.scitotenv.2018.06.154. 30045515

[ref16] Granath G.; Evans C. D.; Strengbom J.; Fölster J.; Grelle A.; Strömqvist J.; Köhler S. J. The impact of wildfire on biogeochemical fluxes and water quality in boreal catchments. Biogeosciences 2021, 18 (10), 3243–3261. 10.5194/bg-18-3243-2021.

[ref17] Caon L.; Vallejo V. R.; Ritsema C. J.; Geissen V. Effects of wildfire on soil nutrients in Mediterranean ecosystems. Earth-Sci. Rev. 2014, 139, 47–58. 10.1016/j.earscirev.2014.09.001.

[ref18] Lopez A. M.; Avila C. C. E.; VanderRoest J. P.; Roth H. K.; Fendorf S.; Borch T. Molecular insights and impacts of wildfire-induced soil chemical changes. Nat. Rev. Earth Environ. 2024, 5 (6), 431–446. 10.1038/s43017-024-00548-8.

[ref19] Vassilev S. V.; Baxter D.; Andersen L. K.; Vassileva C. G. An overview of the composition and application of biomass ash. Part 1. Phase-mineral and chemical composition and classification. Fuel 2013, 105, 40–76. 10.1016/j.fuel.2012.09.041.

[ref20] Yusiharni E.; Gilkes R. Minerals in the ash of Australian native plants. Geoderma 2012, 189–190, 369–380. 10.1016/j.geoderma.2012.06.035.

[ref21] Santín C.; Otero X. L.; Doerr S. H.; Chafer C. J. Impact of a moderate/high-severity prescribed eucalypt forest fire on soil phosphorous stocks and partitioning. Sci. Total Environ. 2018, 621, 1103–1114. 10.1016/j.scitotenv.2017.10.116. 29103642

[ref22] García-Oliva F.; Merino A.; Fonturbel M. T.; Omil B.; Fernández C.; Vega J. A. Severe wildfire hinders renewal of soil P pools by thermal mineralization of organic P in forest soil: analysis by sequential extraction and 31P NMR spectroscopy. Geoderma 2018, 309, 32–40. 10.1016/j.geoderma.2017.09.002.

[ref23] Adachi K.; Dibb J. E.; Scheuer E.; Katich J. M.; Schwarz J. P.; Perring A. E.; Mediavilla B.; Guo H. Y.; Campuzano-Jost P.; Jimenez J. L.; Crawford J.; Soja A. J.; Oshima N.; Kajino M.; Kinase T.; Kleinman L.; Sedlacek A. J.; Yokelson R. J.; Buseck P. R. Fine ash-bearing particles as a major aerosol component in biomass burning smoke. J. Geophys. Res. Atmos. 2022, 127 (2), e2021JD035657 10.1029/2021jd035657.

[ref24] Jahn L. G.; Jahl L. G.; Bland G. D.; Bowers B. B.; Monroe L. W.; Sullivan R. C. Metallic and crustal elements in biomass-burning aerosol and ash: prevalence, significance, and similarity to soil particles. ACS Earth Space Chem. 2021, 5 (1), 136–148. 10.1021/acsearthspacechem.0c00191.

[ref25] Cerrato J. M.; Blake J. M.; Hirani C.; Clark A. L.; Ali A.-M. S.; Artyushkova K.; Peterson E.; Bixby R. J. Wildfires and water chemistry: effect of metals associated with wood ash. Environ. Sci.: Process. Impacts 2016, 18 (8), 1078–1089. 10.1039/C6EM00123H. 27457586

[ref26] Wu Y.; Pae L. M.; Gu C.; Huang R. Phosphorus chemistry in plant ash: examining the variation across plant species and compartments. ACS Earth Space Chem. 2023, 7 (11), 2205–2213. 10.1021/acsearthspacechem.3c00145.

[ref27] Huang R.; Nicholas S.; Wei Z. Thermochemical transformation of calcium during biomass burning and the effects on postfire aqueous dissolution of macronutrients. Environ. Sci. Technol. 2024, 58 (39), 17304–17312. 10.1021/acs.est.4c04820. 39350656 PMC11447901

[ref28] Turpault M. P.; Calvaruso C.; Dincher M.; Mohammed G.; Didier S.; Redon P. O.; Cochet C. Contribution of carbonates and oxalates to the calcium cycle in three beech temperate forest ecosystems with contrasting soil calcium availability. Biogeochemistry 2019, 146 (1), 51–70. 10.1007/s10533-019-00610-4.

[ref29] Prietzel J.; Klysubun W.; Hurtarte L. C. C. The fate of calcium in temperate forest soils: a Ca K-edge XANES study. Biogeochemistry 2021, 152 (2–3), 195–222. 10.1007/s10533-020-00748-6.

[ref30] Ravel B.; Newville M. ATHENA, ARTEMIS, HEPHAESTUS: data analysis for X-ray absorption spectroscopy using. J. Synchrotron Radiat. 2005, 12, 537–541. 10.1107/S0909049505012719. 15968136

[ref31] Hedley M.; Stewart J.; Chauhan B. Changes in inorganic and organic soil phosphorus fractions induced by cultivation practices and by laboratory incubations. Soil Sci. Soc. Am. J. 1982, 46 (5), 970–976. 10.2136/sssaj1982.03615995004600050017x.

[ref32] Costa P.; Manuel J.; Lobo S. Modeling and comparison of dissolution profiles. Eur. J. Pharm. Sci. 2001, 13 (2), 123–133. 10.1016/s0928-0987(01)00095-1. 11297896

[ref33] McBeath A. V.; Smernik R. J.; Schneider M. P. W.; Schmidt M. W. I.; Plant E. L. Determination of the aromaticity and the degree of aromatic condensation of a thermosequence of wood charcoal using NMR. Org. Geochem. 2011, 42 (10), 1194–1202. 10.1016/j.orggeochem.2011.08.008.

[ref34] Zhang K.; Cheng X.; Dang H.; Zhang Q. Biomass:N:K:Ca:Mg:P ratios in forest stands world-wide: biogeographical variations and environmental controls. Global Ecol. Biogeogr. 2020, 29 (12), 2176–2189. 10.1111/geb.13187.

[ref35] Wroblewski R.; Edstrom L. Distribution of sodium, magnesium, chlorine, calcium, potassium, phosphorus and sulfur in Z-bands, I-bands and a-bands in mammalian striated-muscle. Scanning Microsc. 1994, 8 (3), 601–611. 7747159

[ref36] Tolsma D. J.; Ernst W. H. O.; Verweij R. A.; Vooijs R. Seasonal variation of nutrient concentrations in a semi-arid savanna ecosystem in Botswana. J. Ecol. 1987, 75 (3), 755–770. 10.2307/2260204.

[ref37] Perakis S. S.; Sinkhorn E. R.; Catricala C. E.; Bullen T. D.; Fitzpatrick J. A.; Hynicka J. D.; Cromack K. Jr. Forest calcium depletion and biotic retention along a soil nitrogen gradient. Ecol. Appl. 2013, 23 (8), 1947–1961. 10.1890/12-2204.1. 24555320

[ref38] Ranger J.; Gerard F.; Lindemann M.; Gelhaye D.; Gelhaye L. Dynamics of litterfall in a chronosequence of Douglas-fir (Franco) stands in the Beaujolais mounts (France). Ann. For. Sci. 2003, 60 (6), 475–488. 10.1051/forest:2003041.

[ref39] Huang R.; Fang C.; Zhang B.; Tang Y. Transformations of phosphorus speciation during (hydro)thermal treatments of animal manures. Environ. Sci. Technol. 2018, 52 (5), 3016–3026. 10.1021/acs.est.7b05203. 29431994

[ref40] Werner F.; Prietzel J. Standard protocol and quality assessment of soil phosphorus speciation by P K-edge XANES spectroscopy. Environ. Sci. Technol. 2015, 49 (17), 10521–10528. 10.1021/acs.est.5b03096. 26270570

[ref41] Navrotsky A.; Putnam R. L.; Winbo C.; Rosén E. Thermochemistry of double carbonates in the K_2_CO_3_-CaCO_3_ system. Am. Mineral. 1997, 82 (5–6), 546–548. 10.2138/am-1997-5-614.

[ref42] Wotton B. M.; Gould J. S.; McCaw W. L.; Cheney N. P.; Taylor S. W. Flame temperature and residence time of fires in dry eucalypt forest. Int. J. Wildland Fire 2012, 21 (3), 270–281. 10.1071/WF10127.

[ref43] Huang R. X.; Fang C.; Zhang B.; Tang Y. Z. Transformations of phosphorus speciation during (hydro)thermal treatments of animal manures. Environ. Sci. Technol. 2018, 52 (5), 3016–3026. 10.1021/acs.est.7b05203. 29431994

[ref44] Li J.; Posfai M.; Hobbs P. V.; Buseck P. R. Individual aerosol particles from biomass burning in southern Africa: 2, Compositions and aging of inorganic particles. J. Geophys. Res. Atmos. 2003, 108( (D13), ). 10.1029/2002jd002310.

[ref45] Fosca M.; Rau J. V.; Uskokovic V. Factors influencing the drug release from calcium phosphate cements. Bioact. Mater. 2022, 7, 341–363. 10.1016/j.bioactmat.2021.05.032. 34466737 PMC8379446

[ref46] Rahmani M.; Pourmadadi M.; Shakouri S.; Rahdar A.; Díez-Pascual A. M. Novel hydrogel-based gelatin/polyvinyl alcohol/titanium dioxide nanocomposite as a pH-responsive nanocarrier for the controlled release of quercetin in cancer therapy. BioNanoScience 2024, 14, 5286–5296. 10.1007/s12668-024-01411-2.

[ref47] Islam M.; Adams M. A. Mineral content and nutritive value of native grasses and the response to added phosphorus in a Pilbara rangeland. Trop. Grassl. 1999, 33 (4), 193–200.

[ref48] Krieger C.; Calvaruso C.; Morlot C.; Uroz S.; Salsi L.; Turpault M. P. Identification, distribution, and quantification of biominerals in a deciduous forest. Geobiology 2017, 15 (2), 296–310. 10.1111/gbi.12223. 28130812

[ref49] Yue K.; Ni X. Y.; Fornara D. A.; Peng Y.; Liao S.; Tan S. Y.; Wang D. Y.; Wu F. Z.; Yang Y. S. Dynamics of calcium, magnesium, and manganese during litter decomposition in alpine forest aquatic and terrestrial ecosystems. Ecosystems 2021, 24 (3), 516–529. 10.1007/s10021-020-00532-5.

[ref50] Harper A. R.; Santin C.; Doerr S. H.; Froyd C. A.; Albini D.; Otero X. L.; Vinas L.; Perez-Fernandez B. Chemical composition of wildfire ash produced in contrasting ecosystems and its toxicity to Daphnia magna. Int. J. Wildland Fire 2019, 28 (10), 726–737. 10.1071/WF18200.

[ref51] Wu Y.; Pae L. M.; Huang R. Phosphorus chemistry in plant charcoal: interplay between biomass composition and thermal condition. Int. J. Wildland Fire 2023, 33 (1), WF23096 10.1071/wf23096.

[ref52] van Leeuwen T. T.; van der Werf G. R.; Hoffmann A. A.; Detmers R. G.; Rücker G.; French N. H. F.; Archibald S.; Carvalho J. A.; Cook G. D.; de Groot W. J.; Hély C.; Kasischke E. S.; Kloster S.; McCarty J. L.; Pettinari M. L.; Savadogo P.; Alvarado E. C.; Boschetti L.; Manuri S.; Meyer C. P.; Siegert F.; Trollope L. A.; Trollope W. S. W. Biomass burning fuel consumption rates: a field measurement database. Biogeosciences 2014, 11 (24), 7305–7329. 10.5194/bg-11-7305-2014.

[ref53] Klimas K.; Hiesl P.; Hagan D.; Park D. Burn severity effects on sediment and nutrient exports from southeastern forests using simulated rainfall. For. Sci. 2020, 66 (6), 678–686. 10.1093/forsci/fxaa029.

[ref54] Efthimiou N.; Psomiadis E.; Panagos P. Fire severity and soil erosion susceptibility mapping using multi-temporal Earth Observation data: The case of Mati fatal wildfire in Eastern Attica, Greece. Catena 2020, 187, 104320 10.1016/j.catena.2019.104320. 32255894 PMC7001983

[ref55] Wang R.; Balkanski Y.; Boucher O.; Ciais P.; Peñuelas J.; Tao S. Significant contribution of combustion-related emissions to the atmospheric phosphorus budget. Nat. Geosci. 2015, 8 (1), 48–54. 10.1038/ngeo2324.

[ref56] McCullough I. M.; Brentrup J. A.; Wagner T.; Lapierre J.-F.; Henneck J.; Paul A. M.; Belair M.; Moritz M. A.; Filstrup C. T. Fire characteristics and hydrologic connectivity influence short-term responses of north temperate lakes to wildfire. Geophys. Res. Lett. 2023, 50 (16), e2023GL103953 10.1029/2023gl103953.

[ref57] McCullough I. M.; Cheruvelil K. S.; Lapierre J. F.; Lottig N. R.; Moritz M. A.; Stachelek J.; Soranno P. A. Do lakes feel the burn? Ecological consequences of increasing exposure of lakes to fire in the continental United States. Glob. Change Biol. 2019, 25 (9), 2841–2854. 10.1111/gcb.14732. 31301168

[ref58] Barkley A. E.; Prospero J. M.; Mahowald N.; Hamilton D. S.; Popendorf K. J.; Oehlert A. M.; Pourmand A.; Gatineau A.; Panechou-Pulcherie K.; Blackwelder P.; Gaston C. J. African biomass burning is a substantial source of phosphorus deposition to the Amazon, Tropical Atlantic Ocean, and Southern Ocean. Proc. Natl. Acad. Sci. U.S.A. 2019, 116 (33), 16216–16221. 10.1073/pnas.1906091116. 31358622 PMC6697889

