# Supplementary material for: Speciation and Aqueous Dissolution of Macronutrients in Fire Ash: Variation across Ecosystems and the Effects on Nutrient Cycling
Source: Environ Sci Technol. 2024 Dec 24;59(1):454–66. doi: 10.1021/acs.est.4c07101 (PMC11741002; doi:10.1021/acs.est.4c07101)
Supplement: Supplementary file 1 — es4c07101_si_001.pdf [file es4c07101_si_001.pdf]

Supporting Information

Speciation and aqueous dissolution of macronutrients in fire ash: variation across ecosystems and the effects on nutrient cycling

Lingqun Zeng[1], Shyrill F. Mariano[1], Rixiang Huang[1*], Carmen Sánchez-García[2], Cristina Santin[2,3], Jonay Neris[2,4], Kruthika Kumar[5], Chase K. Glenn[5], Omar El Hajj[5], Anita Anosike[5], Joseph O'Brien[6], and Rawad A Saleh[5]

1. Department of Environmental and Sustainable Engineering, University at Albany
1400 Washington Ave, Albany, New York, 12222, USA

2. Centre for Wildfire Research, Department of Geography, Swansea University, Singleton Campus, Swansea, SA2 8PP, UK

3. Biodiversity Research Institute (IMIB; CSIC – Universidad de Oviedo – Principality of Asturias), Mieres, 33600, Spain.

4. Universidad de La Laguna,
Pabellón de Gobierno, C/ Padre Herrera s/n, 38200 La Laguna, Santa Cruz de Tenerife, Spain

5. School of Environmental, Civil, Agricultural and Mechanical Engineering, University of Georgia, Athens, Georgia, 30602, USA

6. USDA Forest Service Southern Research Station, Athens, Georgia, 30602, USA

*Corresponding Author:
Email: rhuang6@albany.edu
Phone: 518-437-4977

Summary: 15 pages, 6 figures, 7 tables

Text S1. Laboratory burning of biomass

The laboratory burnings were conducted as part of the Georgia Wildland-fire Simulation Experiment (G-WISE). The experiments involved constructing fuel beds using biomass collected from two southeast ecosystems: Southeastern US Piedmont Forest (PD) and Coastal Plains (CP). The fuel beds replicated the biomass loadings, proportions, and packing density of the fuel beds observed in the field based on sampling and light detection and ranging (LIDAR) measurements.[1] The PD biomass contained a mix of broadleaved leaf litter and needle and < 3 cm diameter woody stems of Loblolly pine (*Pinus taeda*) and native grasses (*Andropogon spp* and *Schizachyrium spp*). The CP biomass contained a similar mix of braodleaved litter and needles and woody stems (<3cm) of longleaf pine (*Pinus palustris*) and a native grass (*Aristida*). The biomass was conditioned to moisture contents of either 10% to simulate conditions typical for prescribed fires or 4% to simulate conditions typical for drought-induced wildfires. The experiments were conducted in a 990 m^3 burn room. Smoke particulate matter samples were collected through a sampling line extended to an adjacent instrument room on quartz fiber filters at a flow rate of 5 LPM for approximately 30 minutes. After collection, samples were packaged and stored for subsequent analysis.

Text S2. Synchrotron X-ray absorption spectroscopy

Phosphorus spectra were collected between 2100 – 2250 eV, with step sizes of 2 eV for 2100 – 2140 eV, 0.3 eV for 2140.5 – 2190 eV, and 1 eV for 2190 – 2250 eV. Potassium spectra were collected between 3570 – 3710 eV, and the steps were 2 eV for 3570 – 3595 eV, 0.3 eV for 3595 – 3635 eV, and 1 eV for 3635 –3710 eV. Calcium spectra were collected between 4015 – 4200 eV, and the steps were 2.15 eV for 4015 – 4030 eV, 0.3 eV for 4030 – 4065 eV, and 1 eV for 4065 – 4200 eV.

The reference compounds for LCF were selected based on previous findings on minerals identified in biomass ash,[2, 3] principle component analysis (PCA) and target transformation analysis. Principle component analysis (PCA) was first performed to determine the appropriate numbers of components used for LCF, and 4 components were shown to be sufficient (99.5% variance can be explained by the first 4 components). For P, because Ca, K and Mg are the most abundant cations with a metal to P molar ratio >1, their phosphate minerals are the most likely species in ashes. For Ca, various Ca carbonates, Ca-K double carbonates, sulfates, phosphates, and oxides commonly identified in biomass ash were selected. For K, chloride, carbonates, Ca-K double carbonates, and phosphates were selected. Target transformation analysis was performed to shortlist the references.

The reference spectra for LCF of P XANES data included: (1) amorphous Ca phosphate (ACP); (2) hydroxyapatite (HAP); (3) monopotassium phosphate; (4) trimagnesium phosphate octahydrate ($Mg_3(PO_4)_2$), and details of these reference compounds can be found in our previous study.[4, 5] The K reference spectra selected for LCF included: (1) potassium chloride (KCl); (2)

potassium phosphate (K_3PO_4); (3) K-doped calcite; (4) fairchildite. The Ca reference spectra selected for LCF: (1) butschliite; (2) fairchildite; (3) calcite; (4) calcium oxide (CaO); (5) gypsum; and (6) hydroxyapatite. Sources of the K and Ca reference compounds (or spectra) can be found in Table S1.

Linear combination fitting of the P K-edge XANES spectra was performed at the energy range of 2140 – 2190 eV, Ca K-edge XANES spectra at 4032 – 4132 eV, and K K-edge XANES spectra at 3595 – 3670 eV. During LCF of each sample, all the selected reference spectra were included and all combinations were fitted. The fit with the smallest R-factor was identified. All the fits were re-fitted by adding other excluded species, and check if that will improve the fitting. Because the all-combination fitting sometimes resulted in the inclusion of > 4 species or species with <5% abundance, such spectra were refitted by removing the minor species. The goodness of fit was evaluated using the residual factor (R-factor) and the fit with smallest R factor was deemed best fit. The fitting results were presented in Table S2 to Table S4.

Table S1. Reference compounds used for linear combination fitting (LCF) of Ca and K K-edge XANES data.

Compound	Source	Ref.
Butschliite	Equal moles of K_2CO_3 and $CaCO_3$ heated at 750 K in CO_2 atmosphere for 72 hours.	6
Fairchildite	Equal moles of K_2CO_3 and $CaCO_3$ heated at 920 K in CO_2 atmosphere for 72 hours.	6
Calcite	Eisco mineral specimen	
Ca oxide (lime)	Honeywell Fluka	
Hydroxyapatite (HAP)	Preheated (90°C) 100 mL 0.1 mol/L $Ca(CH_3COO)_2$ and 100 mL 0.06M $(NH_4)_2HPO_4$ were mixed and aged at 90 °C for 2 days (mixed solution pH = 5.0), then washed and filtered.	7
Gypsum	Eisco mineral specimen	
Potassium chloride	Thermo Scientific Chemicals	
Potassium phosphate	Thermo Scientific Chemicals	
Potassium doped calcite	Synthetic calcite doped with K (spectrum from Li *et al.*)	8

Table S2. The LCF fitting results for P in different fire residues samples.

**Sample**	**Percentage of P species (%)**				R-factor	χ^2
	**$Mg_3(PO4)_2$**	**KH_2PO_4**	**ACP**	**HAP**		
Boreal forest	24.1	0	25.1	50.8	0.011	1.89
Temperate conifer	20.9	0	35.6	43.5	0.0095	1.61
Heathland (UK)	32.5	10	28.1	29.3	0.010	1.68
Heathland (Spain)	37.2	0	18.9	43.9	0.013	2.42
Savanna	21	19.9	59.1	0	0.0062	0.79
CP-L	21.2	45.5	33.3	0	0.048	4.93
CP-smoke-L	0	29.4	36.8	33.7	0.026	3.03

Table S3. The LCF fitting results for Ca in different fire residues samples.

Sample	Percentage of Ca species (%)						R-factor	χ^2
	**Butschliite**	**Fairchildite**	**Calcite**	**CaO**	**HAP**	**Gypsum**		
Boreal forest	5.3	48	29.8	0	0	16.9	0.0083	0.30
Temperate conifer	0	30.4	45	0	0	24.6	0.0085	0.28
Temperate eucalypt	0	60.2	31	0	0	8.8	0.0069	0.26
Heathland (UK)	40.3	5.7	0	0	54.0	0	0.0033	0.16
Heathland (Spain)	0	23.7	21.1	0	32.4	22.8	0.0038	0.14
Savanna	0	21.1	29.2	0	30.5	19.2	0.0045	0.16
CP-L	0	74	0	0		26	0.0085	0.34
CP-smoke-L	0	57.4	35.9	6.7		0	0.0065	0.27
PD-L	0	75.3	0	5.2		19.5	0.0056	0.22
PD-smoke-L	0	47	4.5	0		48.5	0.022	0.86
PD-smoke-H	0	54.7	45.3	0		0	0.012	0.45

Table S4. The LCF fitting results for K in different fire residues samples.

Sample	Percentage of K species (%)				R-factor	χ^2
	**KCl**	**K_3PO_4**	**K-doped calcite**	**Fairchildite**		
Temperate conifer	0	0	75.5	24.5	0.012	0.25
Boreal forest	0	29.7	50.6	19.7	0.018	0.31
Savanna	30.6	8.7	29.3	31.4	0.009	0.14
Temperate eucalypt	0	0	90.2	9.8	0.015	0.31
Heathland (Spain)	5.2	0	75.1	19.6	0.018	0.32
Heathland (UK)	0	17	65.6	17.4	0.014	0.26
CP-L	7.3	36.3	43.7	12.8	0.024	0.39
PD-L	6.5	16.8	31.8	44.9	0.018	0.27

Table S5. Fitting results (using the Korsmeyer-Peppas model) for the temporal release of Ca, Mg, and K at buffered pH 6.0 and unadjusted pH.

	Sample	pH	no. of points	k (h^{-n})	n	R^2	pH	no. of points	k (h^{-n})	n	R^2
Ca	Boreal forest	6.0	10	1.68	0.065	0.91	8.0	8	0.49	0.19	0.90
	Temperate conifer		10	4.40	0.021	0.95	9.0	8	0.72	0.13	0.98
	Temperate eucalypt		10	0.71	0.04	0.99	10.6	8	0.25	0.20	0.94
	Heathland (UK)		10	0.59	0.029	0.92	6.6	8	--	--	--
	Heathland (Sp.)		10	1.65	0.048	0.94	8.7	8	0.27	0.12	0.95
	Savanna		10	2.03	0.022	0.97	8.2	8	0.083	0.21	0.76
Mg	Boreal forest	6.0	10	0.49	0.087	0.93	8.0	8	0.31	0.16	0.89
	Temperate conifer		10	0.51	0.067	0.99	9.0	8	0.18	0.19	0.98
	Temperate eucalypt		10	0.21	0.061	0.98	10.6	8	0.12	0.15	0.81
	Heathland (UK)		10	0.29	0.084	0.97	6.6	8	0.11	0.06	0.76
	Heathland (Sp.)		10	0.74	0.057	0.96	8.7	8	0.22	0.24	0.98
	Savanna		10	1.56	0.05	0.97	8.2	8	0.17	0.34	0.99
K	Boreal forest	6.0	10	0.18	0.086	0.94	8.0	8	0.16	0.16	0.96
	Temperate conifer		10	0.35	0.051	0.99	9.0	8	0.20	0.21	0.89
	Temperate eucalypt		10	0.05	0.087	0.88	10.6	8	0.027	0.16	0.55
	Heathland (UK)		10	0.34	0.044	0.97	6.6	8	0.12	0.22	0.97
	Heathland (Sp.)		10	1.22	0.033	0.97	8.7	8	0.78	0.09	0.89
	Savanna		10	4.85	0.017	0.94	8.2	8	2.95	0.10	0.96

Table S6. Fire ash loads and the derived total macronutrient load and available macronutrient load for eucalypt forest.

Fire ash loads (kg/ha)	Ref.	Total P (kg/ha)	Available P (kg/ha)	Total Ca (kg/ha)	Available Ca (kg/ha)	Total K (kg/ha)	Available K (kg/ha)	Total Mg (kg/ha)	Available Mg (kg/ha)
5397.75	9	1.67	0.88	56.41	17.14	13.65	9.18	9.98	6.61
4519.00	10	1.40	0.74	47.22	14.35	11.43	7.69	8.36	5.54
6000.00	11	1.86	0.98	62.70	19.05	15.17	10.21	11.09	7.35
16000.00		4.96	2.62	167.20	50.79	40.46	27.22	29.58	19.60
34000.00		10.54	5.57	355.30	107.94	85.97	57.83	62.87	41.65
5992.84	12	1.86	0.98	62.63	19.03	15.15	10.19	11.08	7.34
8431.14		2.61	1.38	88.11	26.77	21.32	14.34	15.59	10.33
4733.15		1.47	0.78	49.46	15.03	11.97	8.05	8.75	5.80
4038.78		1.25	0.66	42.21	12.82	10.21	6.87	7.47	4.95
2350.73		0.73	0.39	24.57	7.46	5.94	4.00	4.35	2.88
5184.85		1.61	0.85	54.18	16.46	13.11	8.82	9.59	6.35
4242.93		1.32	0.69	44.34	13.47	10.73	7.22	7.85	5.20
6450.43		2.00	1.06	67.41	20.48	16.31	10.97	11.93	7.90
7055.90		2.19	1.16	73.73	22.40	17.84	12.00	13.05	8.64
60530.00	13	18.76	9.91	632.54	192.16	153.05	102.96	111.92	74.15

Table S7. Fire ash loads and the derived total macronutrient load and available macronutrient load for savanna.

Fire ash loads (kg/ha)	Ref.	Total P (kg/ha)	Available P (kg/ha)	Total Ca (kg/ha)	Available Ca (kg/ha)	Total K (kg/ha)	Available K (kg/ha)	Total Mg (kg/ha)	Available Mg (kg/ha)
1049.00	14	11.17	6.88	23.65	9.00	50.04	49.93	11.31	7.35
2508.00		26.70	16.45	56.56	21.52	119.63	119.38	27.03	17.58
326.59	15	3.48	2.14	7.36	2.80	15.58	15.54	3.52	2.29
299.37		3.19	1.96	6.75	2.57	14.28	14.25	3.23	2.10
235.87		2.51	1.55	5.32	2.02	11.25	11.23	2.54	1.65
290.30		3.09	1.90	6.55	2.49	13.85	13.82	3.13	2.04
362.87		3.86	2.38	8.18	3.11	17.31	17.27	3.91	2.54
408.23		4.35	2.68	9.21	3.50	19.47	19.43	4.40	2.86
380.00	16	4.05	2.49	8.57	3.26	18.13	18.09	4.10	2.66

976.11	17	10.39	6.40	22.01	8.38	46.56	46.46	10.52	6.84
600.68		6.39	3.94	13.55	5.16	28.65	28.59	6.47	4.21
13.10	18	0.14	0.09	0.30	0.11	0.62	0.62	0.14	0.09
1678.90		17.87	11.01	37.86	14.41	80.08	79.91	18.09	11.77
52.10		0.55	0.34	1.17	0.45	2.49	2.48	0.56	0.37
11.70		0.12	0.08	0.26	0.10	0.56	0.56	0.13	0.08
95.00	19	1.01	0.62	2.14	0.82	4.53	4.52	1.02	0.67
59.00		0.63	0.39	1.33	0.51	2.81	2.81	0.64	0.41
609.00		6.48	3.99	13.73	5.23	29.05	28.99	6.57	4.27
261.00		2.78	1.71	5.89	2.24	12.45	12.42	2.81	1.83
362.00		3.85	2.37	8.16	3.11	17.27	17.23	3.90	2.54
653.00		6.95	4.28	14.73	5.60	31.15	31.08	7.04	4.58
773.00		8.23	5.07	17.43	6.63	36.87	36.79	8.34	5.42
613.00		6.53	4.02	13.82	5.26	29.24	29.18	6.61	4.30
1000.00		10.65	6.56	22.55	8.58	47.70	47.60	10.79	7.01
1011.00		10.76	6.63	22.80	8.68	48.22	48.12	10.91	7.09
826.00		8.79	5.42	18.63	7.09	39.40	39.32	8.91	5.79
490.60	20	5.22	3.22	11.06	4.21	23.40	23.35	5.29	3.44
237.80		2.53	1.56	5.36	2.04	11.34	11.32	2.57	1.67
327.30		3.48	2.15	7.38	2.81	15.61	15.58	3.53	2.29
473.20		5.04	3.10	10.67	4.06	22.57	22.52	5.11	3.32
400	21	4.26	2.62	9.02	3.43	19.08	19.04	4.32	2.80
1300.00		13.84	8.53	29.32	11.16	62.01	61.88	14.03	9.11
2200.00		23.42	14.43	49.61	18.88	104.94	104.72	23.75	15.42
1500.00		15.97	9.84	33.83	12.87	71.55	71.40	16.19	10.52
540.00	22	5.75	3.54	12.18	4.63	25.76	25.70	5.83	3.79
480.00		5.11	3.15	10.82	4.12	22.90	22.85	5.18	3.36
650.00		6.92	4.26	14.66	5.58	31.01	30.94	7.02	4.56
800.00		8.52	5.25	18.04	6.87	38.16	38.08	8.64	5.61
500.00	23	5.32	3.28	11.28	4.29	23.85	23.80	5.40	3.51

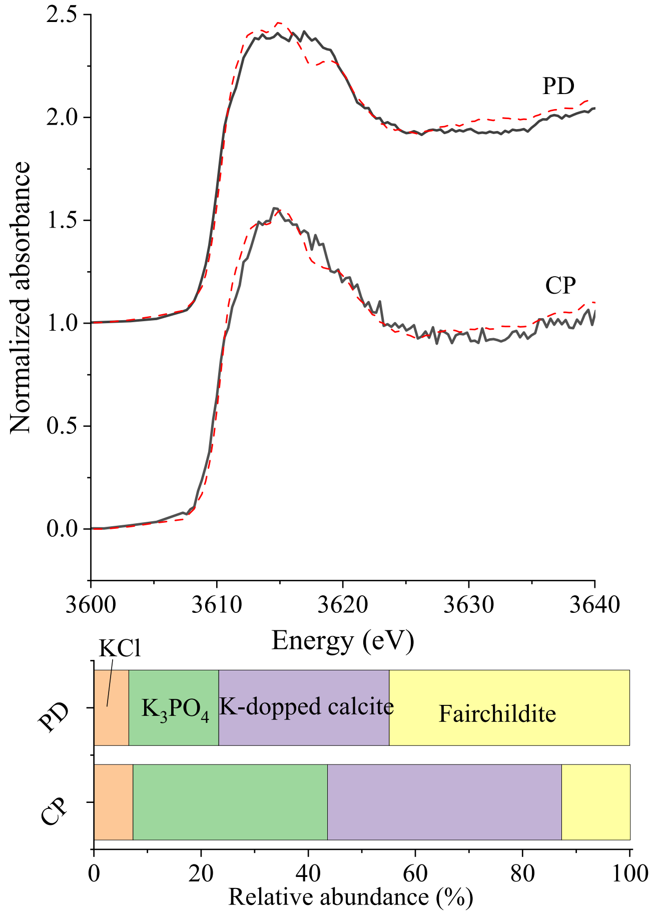

Figure S1. Potassium K-edge XANES spectra and the LCF results of fire ash from simulated burning.

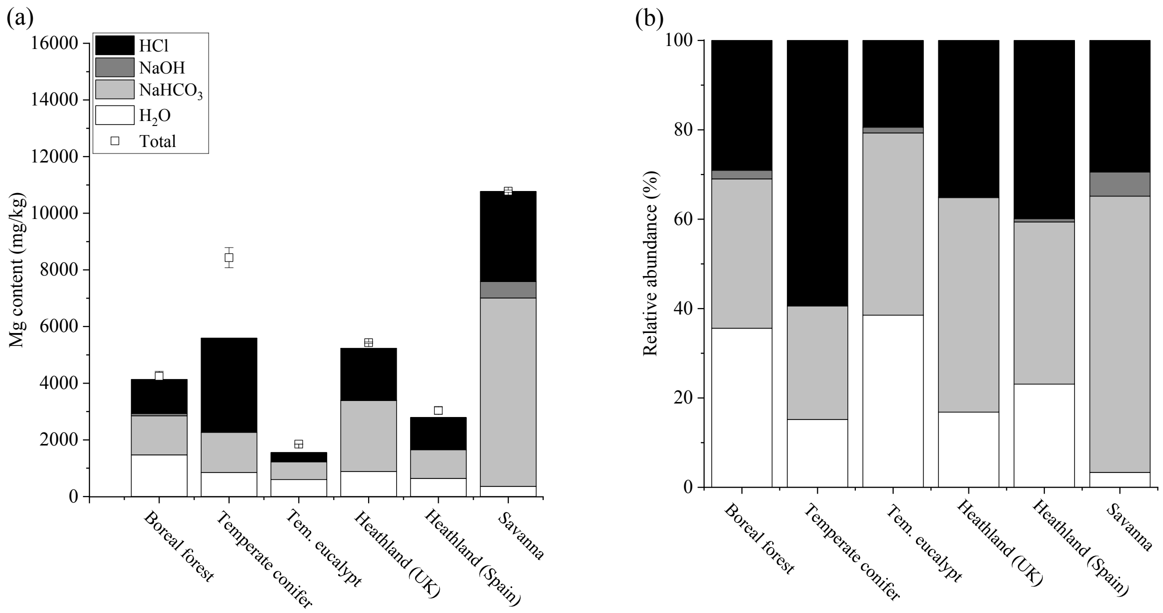

Figure S2. The partitioning of Mg (a – absolute concentration, b – relative abundance) in the four sequential extraction pools by modified Hedley extraction.

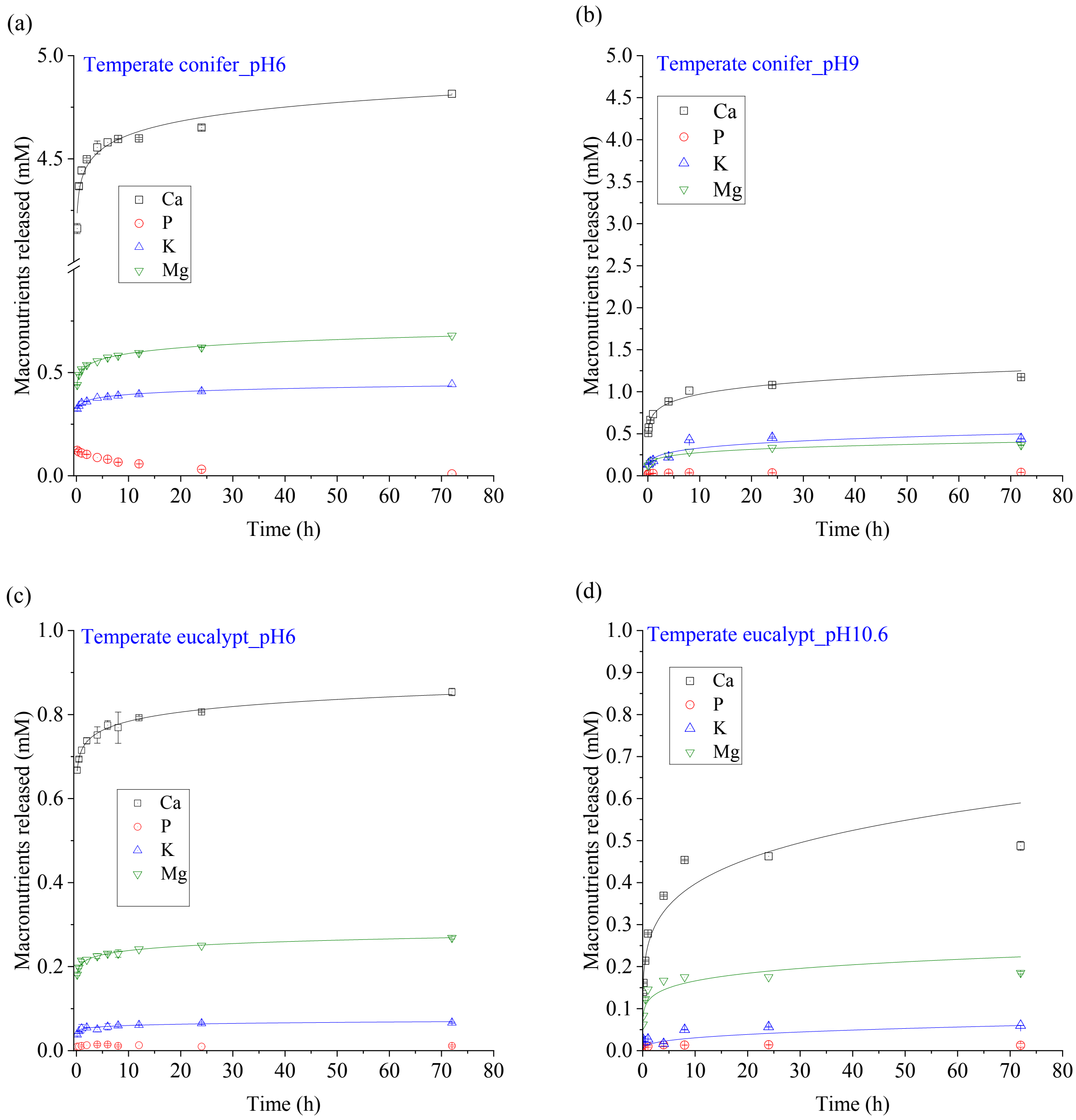
(a)
Temperate conifer_pH6
Ca
P
K
Mg
Macronutrients released (mM)
Time (h)
(b)
Temperate conifer_pH9
Ca
P
K
Mg
Macronutrients released (mM)
Time (h)
(c)
Temperate eucalypt_pH6
Ca
P
K
Mg
Macronutrients released (mM)
Time (h)
(d)
Temperate eucalypt_pH10.6
Ca
P
K
Mg
Macronutrients released (mM)
Time (h)

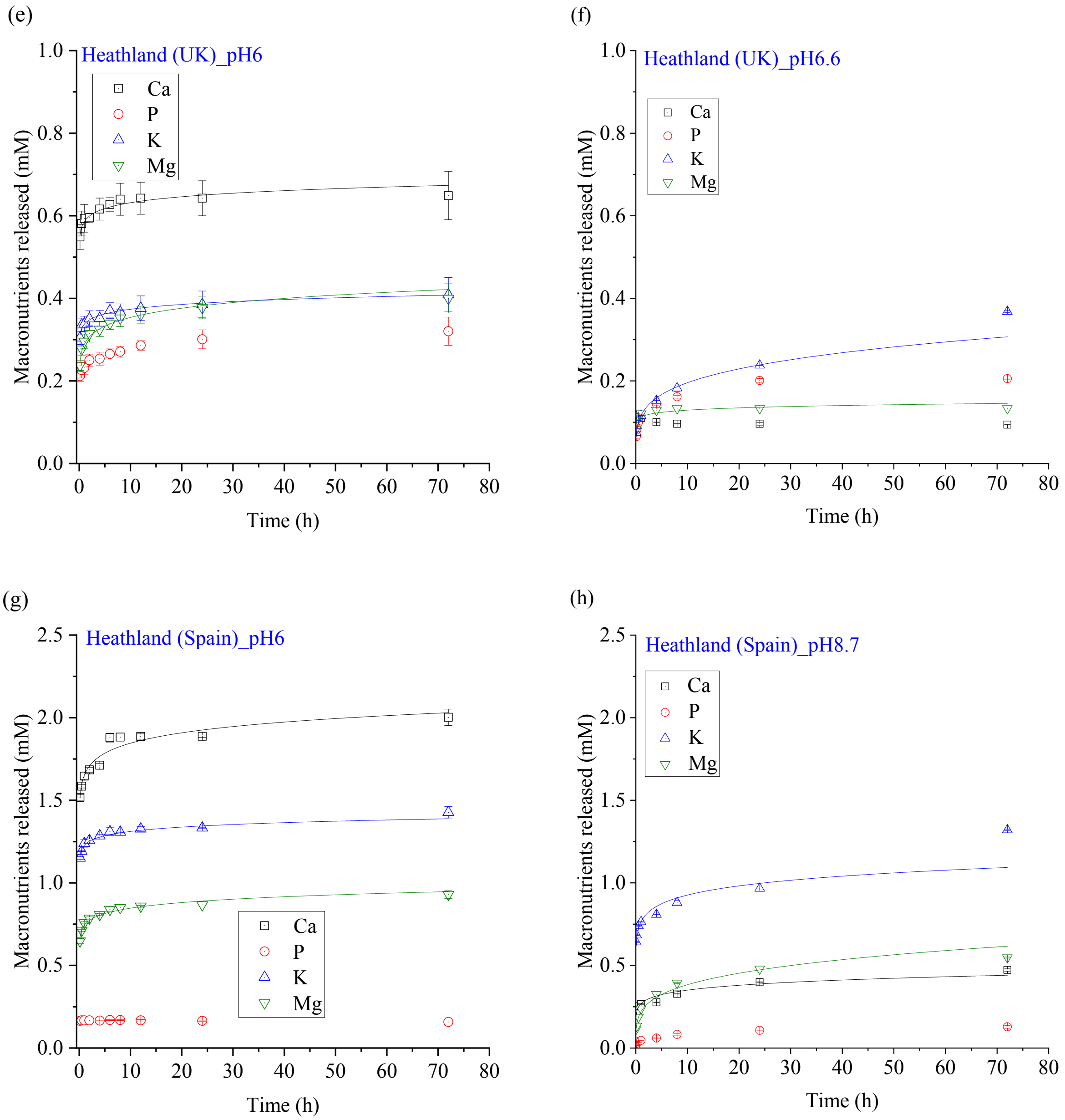

Figure S3. Release kinetics of Ca, K, Mg and P for fire residues from different ecosystems with 200 mg ash/40 mL solution at pH 6.0 (left)and at pH without adjustment (right). The solid lines were curve fitting with the Korsmeyer–Peppas model.

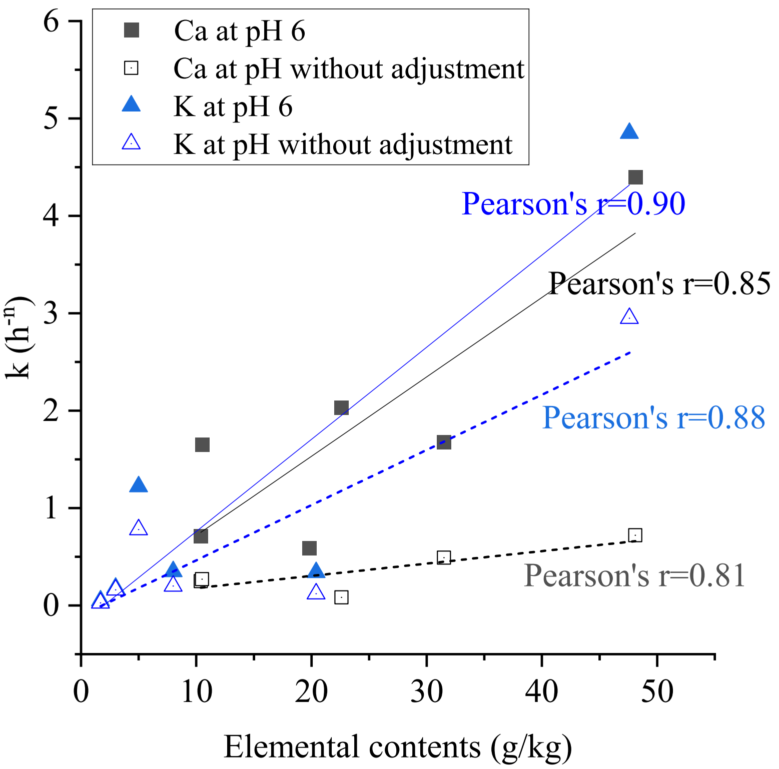

Figure S4. Correlation between the Korsmeyer–Peppas model rate constant *k* values and total elemental content in ash for Ca and K at the buffered and unadjusted pH.

References

1. Loudermilk, E. L.; O'Brien, J. J.; Mitchell, R. J.; Cropper, W. P.; Hiers, J. K.; Grunwald, S.; Grego, J.; Fernandez-Diaz, J. C., Linking complex forest fuel structure and fire behaviour at fine scales. *Int J Wildland Fire* **2012,** *21*, (7), 882-893.
2. Vassilev, S. V.; Baxter, D.; Andersen, L. K.; Vassileva, C. G., An overview of the composition and application of biomass ash. Part 1. Phase-mineral and chemical composition and classification. *Fuel* **2013,** *105*, 40-76.
3. Yusiharni, E.; Gilkes, R., Minerals in the ash of Australian native plants. *Geoderma* **2012,** *189-190*, 369-380.
4. Wu, Y.; Pae, L. M.; Gu, C.; Huang, R., Phosphorus Chemistry in Plant Ash: Examining the Variation across Plant Species and Compartments. *ACS Earth and Space Chemistry* **2023,** *7*, (11), 2205-2213.
5. Huang, R.; Nicholas, S.; Wei, Z., Thermochemical Transformation of Calcium during Biomass Burning and the Effects on Postfire Aqueous Dissolution of Macronutrients. *Environ Sci Technol* **2024,** *58*, (39), 17304-17312.
6. Navrotsky, A.; Putnam, R. L.; Winbo, C.; Rosén, E., Thermochemistry of double carbonates in the K2CO3-CaCO3 system. *American Mineralogist* **1997,** *82*, (5-6), 546-548.
7. Huang, R. X.; Fang, C.; Zhang, B.; Tang, Y. Z., Transformations of Phosphorus Speciation during (Hydro)thermal Treatments of Animal Manures. *Environ Sci Technol* **2018,** *52*, (5), 3016-3026.
8. Li, W.; Liu, X.-M.; Hu, Y., Potassium and Calcium K-Edge XANES in Chemical Compounds and Minerals: Implications for Geological Phase Identification. *Geostandards and Geoanalytical Research* **2020,** *44*, (4), 805-819.
9. Santín, C.; Otero, X. L.; Doerr, S. H.; Chafer, C. J., Impact of a moderate/high-severity prescribed eucalypt forest fire on soil phosphorous stocks and partitioning. *Science of the Total Environment* **2018,** *621*, 1103-1114.
10. Raison, R.; Khanna, P.; Woods, P., Transfer of elements to the atmosphere during low-intensity prescribed fires in three Australian subalpine eucalypt forests. *Canadian Journal of Forest Research* **1985,** *15*, (4), 657-664.
11. Santín, C.; Doerr, S. H.; Otero, X. L.; Chafer, C. J., Quantity, composition and water contamination potential of ash produced under different wildfire severities. *Environmental Research* **2015,** *142*, 297-308.
12. Jenkins, M. E.; Bell, T. L.; Poon, L. F.; Aponte, C.; Adams, M. A., Production of pyrogenic carbon during planned fires in forests of East Gippsland, Victoria. *Forest Ecology and Management* **2016,** *373*, 9-16.
13. Mastrolonardo, G.; Hudspith, V. A.; Francioso, O.; Rumpel, C.; Montecchio, D.; Doerr, S. H.; Certini, G., Size fractionation as a tool for separating charcoal of different fuel source and recalcitrance in the wildfire ash layer. *Science of the total environment* **2017,** *595*, 461-471.
14. Kauffman, J. B.; Cummings, D.; Ward, D., Relationships of fire, biomass and nutrient dynamics along a vegetation gradient in the Brazilian cerrado. *Journal of ecology* **1994**, 519-531.
15. Pivello, V. R.; Coutinho, L. M., Transfer of macro-nutrients to the atmosphere during experimental burnings in an open cerrado (Brazilian savanna). *Journal of tropical ecology* **1992,** *8*, (4), 487-497.
16. Cook, G., The fate of nutrients during fires in a tropical savanna. *Australian Journal of Ecology* **1994,** *19*, (4), 359-365.

17. N'Dri, A. B.; Konan, L. N., Does the date of burning affect carbon and nutrient losses in a humid savanna of West Africa. *Environment and Natural Resources Research* **2018,** *8*, (3), 1927-0496.
18. Rossiter-Rachor, N.; Setterfield, S.; Douglas, M.; Hutley, L.; Cook, G., Andropogon gayanus (gamba grass) invasion increases fire-mediated nitrogen losses in the tropical savannas of northern Australia. *Ecosystems* **2008,** *11*, 77-88.
19. Shea, R. W.; Shea, B. W.; Kauffman, J. B.; Ward, D. E.; Haskins, C. I.; Scholes, M. C., Fuel biomass and combustion factors associated with fires in savanna ecosystems of South Africa and Zambia. *Journal of Geophysical Research: Atmospheres* **1996,** *101*, (D19), 23551-23568.
20. Barbosa, R. I.; Fearnside, P. M., Above-ground biomass and the fate of carbon after burning in the savannas of Roraima, Brazilian Amazonia. *Forest Ecology and Management* **2005,** *216*, (1-3), 295-316.
21. De Castro, E. A.; Kauffman, J. B., Ecosystem structure in the Brazilian Cerrado: a vegetation gradient of aboveground biomass, root mass and consumption by fire. *Journal of tropical ecology* **1998,** *14*, (3), 263-283.
22. Lacaux, J.; Cachier, H.; Delmas, R., Biomass burning in Africa: An overview of its impact on atmospheric chemistry. *Fire in the environment: The ecological, atmospheric, and climatic importance of vegetation fires* **1993**, 159-191.
23. Hopkins, B., Observations on savanna burning in the Olokemeji Forest Reserve, Nigeria. *Journal of Applied Ecology* **1965**, 367-381.
